# Supplementary material for: Genome-wide gene expression noise in Escherichia coli is condition-dependent and determined by propagation of noise through the regulatory network
Source: PLoS Biol. 2021 Dec 17;19(12):e3001491. doi: 10.1371/journal.pbio.3001491 (PMC8719677; doi:10.1371/journal.pbio.3001491)
Supplement: S1 Text — (PDF) [file pbio.3001491.s001.pdf]

S1 Supporting Information

Contents

S1 Supporting Information **1**
S1.1 Supplementary Methods and Texts . . . 1
S1.1.1 Strains . . . 1
S1.1.2 Growth conditions . . . 1
S1.1.3 Flow cytometry quantification of fluorescence . . . 2
S1.1.4 Time-course quantification of fluorescence . . . 2
S1.1.5 Minimal variance as a function of mean and noise estimation . . . 2
S1.1.6 Relation between noise and regulatory inputs . . . 3
S1.1.7 Fitting noise in terms of regulatory inputs . . . 4
S1.1.8 TFs that propagate noise in specific growth conditions . . . 5
S1.1.9 TFs that propagate noise in all growth conditions . . . 6
S1.1.10 Principal component analysis . . . 6
S1.1.11 Comparing noise levels of constitutive synthetic promoters with regulated native promoters . . . 7
S1.1.12 Impact of plasmid copy numbers on mean and noise levels . . . 7
S1.1.13 Information on the growth conditions. . . . 9
S1.2 Supplementary Figures . . . 10

S1.1 Supplementary Methods and Texts

S1.1.1 Strains

All 1810 strains were taken from [1]. In short, each strain carries a transcriptional fusion of a given native *E.coli* promoter followed by a strong ribosomal binding site and *gfp-mut2* (a fast maturing GFP) on a low copy-number plasmid (pUA66 or pUA139 with pSC101 origin, $\sim$ 6 copies per cell). The library contains a construct for $\sim$75% of all intergenic regions longer than 40bp in *E.coli*'s genome flanked by 50 (resp. 150bp) of the downstream (resp. upstream) sequence in order to include most regulatory interactions found on the chromosome.

S1.1.2 Growth conditions

The strain library was stored at -80° C in LB + 7.5% glycerol in microtiter plates. Individual plates were inoculated into fresh media of interest (200 μl) and incubated for two overnights in the same condition before fluorescence measurements. Dilutions ($\sim$1/2000) between overnights were done using a 96 Solid Pin Replicator (V&P, 409). The library was grown in a total of 8 different conditions: minimal media, M9 (0.1mM $CaCl_2$, 1mM $MgSo_4$, 1 x M9 salts [Sigma M6030]) supplemented with either 0.2% glucose (w/v), 0.2% glycerol (v/v), 0.2% lactose (w/v), 0.4M NaCl (+ 0.2% glucose [w/v]) or 1.5 ng/ml ciprofloxacin (+ 0.2% glucose [w/v]); a MOPS based synthetic rich media (Teknova, M2105) supplemented with 0.2% glucose, and two stationary phase conditions, where plates were grown for either 16h or 30h in M9 minimal media + 0.2% glucose (w/v). Note that optical density typically saturates after about 10 hours of growth in these conditions (Fig A).

All media, except the one containing ciprofloxacin, were supplemented with 50 μg/ml kanamycin. The overnights for the sub-MIC ciprofloxacin condition were done in M9 glucose 0.2%, and only at the day of quantification ciprofloxacin was added. On the quantification day, cells were diluted between 200 and 1000-fold depending on the condition (C Table) and grown until mid-exponential phase at 37°, shaken at 600rpm. Growth rates were estimated independently for individual strains in each condition by monitoring

the optical density (OD_{600}) every 90s during 15-25 hours at 37°C in a plate reader (Biotek Synergy 2). We defined the growth rate α as the slope of a straight-line fit of $\log(OD_{600})$ against time.

To estimate cell sizes, a strain of the library containing a plasmid without promoter was selected and grown as described. Cells were then placed on a 1% agarose pad and phase contrast images were obtained with a Nikon Ti-E microscope using a 100× Ph3 objective (NA 1.45) and an Hamamatsu Orca-Flash 4.0 v2 camera. Cell outlines were identified using a custom MATLAB pipeline.

S1.1.3 Flow cytometry quantification of fluorescence

We measured the distribution of GFP fluorescence levels in single cells using a FACSCanto II (BD Biosciences) with a high-throughput sampler (HTS), fluorescence excitation at 488 nm and a 530/30 nm filter for emission. For each strain we collected 5×10^4 events. We used a Bayesian procedure that removes outliers to extract the mean and variance of the log-fluorescence distributions as described in [2]. Briefly, we first fitted the 4-dimensional signal distribution of forward and side scatter heights and widths by a mixture of a multi-variate Gaussian and a uniform 'background' distribution. For each event, we then calculated the posterior probability that it derives from the central multi-variate Gaussian, and all events with lower than 50% posterior probability were removed. For the remaining cells, the logarithms of the fluorescence signals (logarithm of the height of the peak) were fitted to a mixture of a Gaussian and a uniform background distribution. That is, the probability of observing log-fluorescence y has the form:

$$P(y|\mu, \sigma, \rho) = \frac{\rho}{\sqrt{2\pi}\sigma} e^{-\frac{(y-\mu)^2}{2\sigma^2}} + \frac{1-\rho}{\Delta}, \tag{1}$$

where μ and σ^2 are the mean and variance of the log-fluorescence distribution, ρ is the fraction of cells deriving from the Gaussian, and $\Delta = y_{\max} - y_{\min}$ is the range of observed log-fluorescence values. Given n single-cell log-fluorescence measurements y_1, y_2, ..., y_n for a given promoter in a given condition, the likelihood is simply given by $L(\mu, \sigma, \rho) = \prod_{i=1}^{n} P(y_i|\mu, \sigma, \rho)$ and we fit μ, σ, and ρ by maximizing this likelihood. The data processing method of [2] is available as an R package at (`https://github.com/vanNimwegenLab/vngFCM.git`).

In order to assess reproducibility of the measurements, we measured a subset of the library on multiple days and estimated means and variances of each promoter separately for each day (Fig D). We defined the mean and variance of each promoter that was measured more than once as the average over its replicates. For each of the individual promoters shown in Fig 2 of the main text, 6 independent measurements were taken.

S1.1.4 Time-course quantification of fluorescence

One of the plates of the library (95 strains) was grown in M9 + 0.4M NaCl during two overnights. At the day of the quantification a 1/200 dilution was done in 1ml of fresh media in a 96 deep-well plate (with 1 glass-bead per well for better shaking). The plate was covered with a breathable sealing film and grown at 37°, shaken at 600 rpm. At 9 consecutive time points after dilution (after 0h, 1h, 2h, 3h, 5h, 6.5h, 8.5h, 10h and 11h), 100 μl of the culture was transferred into a 96-well plate and used for fluorescence quantification.

S1.1.5 Minimal variance as a function of mean and noise estimation

Flow cytometry measurements show a clear lower bound on noise levels (variance of log-fluorescence) that depends on the mean of expression. In previous work [3] we derived a functional form for this noise floor as a function of mean expression which takes into account that total fluorescence is a sum of background fluorescence and fluorescence deriving from GFP, and that the variance in GFP levels is a sum of a 'Poissonian' term that is proportional to mean fluorescence, and a 'multiplicative' term proportional to

mean fluorescence squared. If we denote the background fluorescence in condition c by $f_{bg,c}$ and the average fluorescence of promoter p in condition c by $\langle f_{p,c} \rangle$, the minimal variance in log-fluorescence takes the form

$$\sigma^2_{\min}\left(\langle f_{p,c}\rangle\right) = a_c \left(1 - \frac{f_{bg,c}}{\langle f_{p,c}\rangle}\right)^2 + \frac{b_c}{\langle f_{p,c}\rangle}\left(1 - \frac{f_{bg,c}}{\langle f_{p,c}\rangle}\right), \qquad (2)$$

where b_c is the prefactor of the 'Poissonian' component of the variance proportional to the mean, and a_c is the prefactor of the component of the variance proportional to the square of the mean.

We estimated the average background fluorescence in each condition from plasmids without a promoter upstream of *gfp-mut2* that were included in each individual plate. As discussed in [2], the above model breaks down in the regime where promoters display fluorescence levels close to background fluorescence, we only considered promoters with mean larger than $2f_{bg,c}$ for further analysis. We fitted the following parameters for the minimal variance in each condition:

Table A. Comparison of the fitted lowerbound parameters in the different conditions

Condition	a_c	b_c	$f_{bg,c}$
Synthetic Rich	0.015	410	180
Ciprofloxacin 1.5ng/ml	0.05	570	230
M9 glucose	0.05	530	220
M9 lactose	0.063	550	220
M9 glycerol	0.065	580	205
M9 0.4M NaCl	0.065	500	205
Stationary phase 16h	0.075	600	190
Stationary phase 30h	0.075	600	190

Table B. Percentage of promoters above background (i.e. $2f_{bg,c}$).

Condition	%
Synthetic Rich	40.0
Ciprofloxacin 1.5ng/ml	58.5
M9 glucose	54.6
M9 lactose	57.2
M9 glycerol	59.0
M9 0.4M NaCl	51.2
Stationary phase 16h	58.2
Stationary phase 30h	58.9

To obtain a noise level for each promoter that does not systematically depend on mean, we defined the noise N_{pc} of promoter p in condition c as the difference between the measured variance and the fitted minimal variance:

$$N_{pc} = \sigma^2_{pc} - \sigma^2_{\min}\left(\langle f_{p,c}\rangle\right). \qquad (3)$$

S1.1.6 Relation between noise and regulatory inputs

We used the same promoter annotation as in Wolf et al. 2015, where the promoter fragments had been re-annotated by mapping the primer pairs used to construct the library to the *E.coli* K12 MG1655 genome. From all measured promoters we were able to annotate 94% unambiguously to an immediately downstream

gene. We obtained all gene-TF regulation annotations from RegulonDB [4] and counted for each gene the number of unique transcription factors known to regulate it.

We sorted all annotated genes by their average noise across all conditions ($\bar{N}_p$) and as a function of a cut-off in $\bar{N}_p$, we calculated the mean and standard-error of the number of regulatory inputs of all genes with $\bar{N}_p$ values above the cut-off. We also performed this analysis separately in each of the conditions c, using the noise levels N_{pc} as opposed to the averages (Fig L). To estimate the statistical significance of the relationship between noise and number of regulatory inputs, we calculated for each cut-off in noise (for both $\bar{N}_p$ and N_{pc}) a t-statistic of the difference in mean number of regulatory inputs between promoters with noise above and below the cut-off:

$$t = \frac{\mu_a - \mu_b}{\sqrt{\frac{v_a}{n_a} + \frac{v_b}{n_b}}},$$

where μ_a and μ_b are the average number of regulatory inputs for promoters with noise above and below the cut-ff, v_a and v_b the variances in numbers of regulatory inputs for promoters above and below the cut-off, and n_a and n_b the number of promoters above and below the cut-off in noise (Fig M).

We also calculated, for each cut-off value in noise, the fraction (f) of promoters above the cut-off with at least one regulatory input annotated. Both for the average cut-off value in noise across conditions ($\bar{N}_p$) and for a cut-off value in noise for each condition separately (N_{pc}, Fig P). In Fig 4B in the main text and Fig P the standard-error for these fractions f at each cut-off value was calculated as:

$$\sqrt{\frac{f(1-f)}{n}},$$

with n being the total number of promoters above the cut-off.

As a measure of noise plasticity of each promoter p, we calculated the variance of the noise levels N_{pc} across conditions. As a measure of expression plasticity of each promoter p, we calculated the variance of the mean expression level across conditions.

S1.1.7 Fitting noise in terms of regulatory inputs

To model noise in terms of regulatory inputs we adapted a method, called Motif Activity Response Analysis, which models gene expression levels in terms of computationally predicted regulatory sites in promoters and condition-dependent activities of regulators using a linear model [5, 6].

As explained in the main text, we model the noise N_{pc} of each promoter p in each condition c as a linear function of the condition-dependent noise-propagating activities A_{rc} of the regulators known to regulate promoter p :

$$(N_{pc} - \bar{N}_c) = \epsilon + \sum_r (S_{pr} - \bar{S}_r) A_{rc}, \tag{4}$$

where $\bar{N}_c$ is the average noise level of all promoters in condition c, and ϵ is a noise term that is assumed Gaussian distributed with mean 0 and unknown variance. We used the RegulonDB database [4] to set a binary matrix of known regulatory inputs, i.e. S_{pr} is 1 when promoter p is known to be regulated by TF r and 0 otherwise. In addition $\bar{S}_r$ is the average of S_{pr} across all promoters, i.e. the fraction of promoters targeted by regulator r. Note that for this analysis we also included binding of sigma factors a source of regulation.

The noise term ϵ, which reflects the deviation between the measurements and our simple model, is assumed to be Gaussian distributed with mean zero and unknown variance. To avoid overfitting, the model also includes a Gaussian prior over noise-propagation activities A_{rc} that has mean zero and a variance that is set using cross-validation. In particular, maximal posterior probability noise-propagation

activities A_{rc} are inferred on 80% of the promoters, and the variance of the prior is set so as to minimize the squared-error of the predictions on the remaining 20% of the promoters. Thus, a different prior is fitted for each condition. As a simple measure of the quality of the fit, we used the fraction of the total variance in the data that is explained by the model (FOV).

For each regulator and condition, we obtain the full posterior distribution over the noise-propagation activity A_{rc} and use the standard-deviation δA_{rc} of this posterior as an error-bar for the inferred activity A_{rc}. In addition, we use the z-like statistic $z_{rc} = A_{rc}/\delta A_{rc}$ as a measure of significance of regulator r in condition c.

We defined the average noise-propagating strength $\bar{A}_r$ of each regulator r as a weighted average over the 8 conditions:

$$\bar{A}_r = \frac{\sum_c \frac{A_{rc}}{\delta A_{rc}^2}}{\sum_c \frac{1}{\delta A_{rc}^2}}, \tag{5}$$

and the corresponding error-bar $\delta \bar{A}_r$ as

$$\delta \bar{A}_r = \frac{1}{\sqrt{\sum_c \frac{1}{\delta A_{rc}^2}}}. \tag{6}$$

Finally, the average significance $\bar{z}_r$ of motifs over all conditions was then estimated as:

$$\bar{z}_r = \frac{\bar{A}_r}{\delta \bar{A}_r}. \tag{7}$$

Note that, roughly speaking, z_r corresponds to the number of standard-deviations the activity of regulator r is away from zero on average.

To confirm the significance of our fits, we performed tests in which we randomly shuffled the rows of the noise-level matrix N_{pc}, thereby randomizing the association between noise levels and regulatory inputs. We fitted the model to this randomized data and found consistently low FOVs (Fig 4C in main text, yellow bars).

S1.1.8 TFs that propagate noise in specific growth conditions

Besides the TFs LexA and FlhDC discussed in the main text, our analysis predicted ArcA, CytR, and Fur to each significantly propagate noise in only one growth condition. The TF ArcA only significantly propagates noise in the condition with M9 + 0.4M NaCl. ArcA is a general regulator that controls the aerobic/anaerobic expression of respiratory proteins and diverges metabolism into fermentation [7]. Under salt stress major adaptations in metabolism occur and fermentation products increase [8], which is consistent with heterogeneous activity of ArcA in these conditions.

The TF CytR was found to contribute to noise propagation only in late stationary phase. CytR regulates genes involved in nucleoside uptake and utilization [9] and it was recently found that mutations in CytR have a fitness advantage during long term stationary phase [10], which was hypothesized to result from an increased ability to import and use nucleosides that occur in the stationary phase environment due to cell death. Heterogeneity in CytR activation late in stationary phase is consistent with this functional role.

Finally, the TF Fur, which regulates genes involved in iron homeostasis [11], had significant noise propagating activity only in the M9 + 0.2% lactose condition. In contrast to the other four cases, we do not have an obvious biological interpretation for why Fur activity might be especially heterogeneous during growth on lactose.

S1.1.9 TFs that propagate noise in all growth conditions

The most significant condition-independent noise propagating factor was H.NS, a general transcriptional repressor that regulates around 5% of all *E.coli* promoters. It belongs to the family of 'nucleoid associated' proteins, acting as a histone-like molecule by binding to curved DNA and inhibiting transcription [12]. The second most significant noise propagating TF is the sigma factor Sigma38 (*rpoS*), which is considered the central regulator of gene expression in early stationary phase and under environmental stress [13, 14]. It has been established that, in contrast to rich media, *rpoS* levels in minimal media (the basis of 7 of our 8 conditions) are also high during exponential phase [15]. Moreover, it was recently shown that *rpoS* activity is heterogeneous among single cells in M9 glucose [16].

Two further significant condition-independent noise propagators were CRP and PhoB. CRP is a global regulator of genes involved in carbon source catabolism and its activity has been proposed to reflect carbon source influx [17]. PhoB regulates the response to inorganic phosphate (Pi) starvation and binds to the DNA as a dimer after being phosphorylated by a histidine kinase (PhoR) under Pi limited conditions [18]. We hypothesize that, in our growth conditions, both carbon source influx and Pi concentration are sufficiently limiting that cell-to-cell fluctuations cause significant fluctuations in the activities of CRP and PhoB.

Finally, the two last factors that were found to be significantly contributing to noise propagation across all conditions (GadX and GadW), belong to a family of regulators involved in the response to acid stress [19]. The appearance of these factors may also be explained by our experimental setup. Oxygen levels in microtiter plates can easily become limiting and this oxygen deprivation leads to production of fermentation products [7], even when oxygen is still present [20]. Fermentation products are known to acidify the medium [21], which can activate the response to acid stress. Moreover, the fact that we find GadX and GadW as noise propagators is consistent with a recent publication, where it was shown that heterogeneous expression of the gadBC operon (heavily regulated by GadX and GadW) correlated with single-cell survival to high acid stress induced by an antibiotic [22].

S1.1.10 Principal component analysis

For each promoter we gathered a list of 10 features associated with the immediately downstream gene using both the measurement in this study as well as previously published data. In particular we obtained for each promoter:

1. Average RNA level (data taken from [23]).
2. Average protein level (data taken from [24]).
3. Fraction of optimal codons (data taken from [25]).
4. Substitution rate at synonymous sites dS (data taken from [25]).
5. Substitution rate at non-synonymous sites dN (data taken from [25]).
6. Average of the mean in log-expression across conditions (this study).
7. Expression plasticity, i.e. variance of the mean in log-expression across conditions (this study).
8. Average of the promoter noise across conditions (this study).
9. Noise plasticity, i.e. variance of the promoter noise across conditions (this study).
10. Number of regulatory inputs (data taken from [4]).

Using these measurements, we calculated a covariance matrix containing all the variances of each of these features across genes, and the covariances of each pair of features. Note that not all features were available for all genes so that, for each pair of features, we estimated the covariance from the set of genes for which both features were available. We then normalized the covariance matrix by dividing each entry C_{ij} by the square-root of the product of variances, i.e. $C_{ij} \to R_{ij} = C_{ij}/\sqrt{C_{ii}C_{jj}}$, turning it into a matrix of Pearson correlation coefficients. We then performed PCA on this correlation matrix. Finally, for the first two principal components we calculated what fraction of the principal vector's length was accounted for by each feature.

S1.1.11 Comparing noise levels of constitutive synthetic promoters with regulated native promoters

We randomly selected a set of synthetic promoters (72 expressing at a medium level and 72 expressing at a high level) from the library of constitutive promoters evolved in [3]. In short, each synthetic promoter is the result of the assembly of random nucleotides of around 100-150bp ligated into the plasmid puA66. The sequences were evolved to two predefined expression levels (medium and high), after 5 rounds of FACS sorting and PCR mutagenesis.

We grew the set of selected synthetic promoters in four of our conditions: minimal media (M9) supplemented with 0.2% glucose, 0.2% lactose, 0.2% glycerol and 0.4M NaCl (+0.2% glucose) with appropriate antibiotics (50μg/ml Kanamycin), measured the distribution of single-cell log-fluorescence levels, and estimated mean expression and expression noise levels, as described previously in the methods section.

To compare mean expression and noise levels of regulated versus constitutive promoters we selected, from the measured library of native promoters [1], those promoters with at least 1 annotated binding site in RegulonDB [4] beyond the sigma site.

We estimated for each library (native and synthetic) the average expression noise level across the four conditions (average noise), as well as the variance in noise and expression across the four conditions (expression and noise plasticity). We compared each of these variables between the libraries as shown in Fig O .

S1.1.12 Impact of plasmid copy numbers on mean and noise levels

To assess the contribution of potential plasmid copy number fluctuations on noise levels, and in particular on the noise floor, we measured and compared means and noise levels displayed by promoters that were either on the pUA66 plasmid or integrated in the chromosome.

First, we selected a set of promoters characterized by noise levels close to the noise floor in one of our conditions (M9 + 0.2 % glucose): 5 native promoters (regulating *alaS, gshA, ybhC, ygaZ, yidC*) and 4 synthetic promoters (two high expressing and two medium expressing, which we denote high 1, high 2, medium 1, and medium 2) from [3]. We also added one synthetic promoter with higher expression noise (denoted medium 3, also from [3]) to this list.

Second, we integrated the pUA66 expression cassette within the chromosome of *E. coli* MG1655. The pUA66 plasmids containing the selected promoters were purified using standard column purification protocols and the expression cassettes (promoter region and *gfp-mut2*) where PCR amplified using the following forward and reverse primers: ATCGGAATTCCGGCAAGAAAGCCATCCAGT and AAAACT-GCAGGGACATTTATTTGTACAATTCATCCATACCAT. The obtained PCR products were ligated into HK022 pOSIP chromosome integration plasmid, using available EcoRI and PstI restriction sites. The whole expression cassettes were then integrated within *E. coli*'s MG1655 chromosome following the pOSIP integration protocol detailed in [26]. Successful integration of a single copy of the expression cassette at the HK022 site was confirmed by PCR and Sanger sequencing for each promoter.

This resulted in chromosomally integrated reporter strains, that could be compared to the original plasmid reporter strains. Plasmid and chromosomally integrated reporter strains were streaked on LB-Agar plates from glycerol stocks and single colonies were used to inoculate M9 + 0.2% glucose overnight cultures (with/without kanamycin for the plasmid/chromosomal strains). Overnight cultures were diluted a 100 fold in the morning, and cells were collected in exponential phase (OD ~ 0.2) for inoculation into a microfluidic device flown with the same media (M9 + 0.2% glucose). Cell size (l) and total fluorescence intensity of GFP (g) were measured and analysed using protocols published previously [27]. For each strain, cell size and GFP intensity were sampled randomly once per cell cycle, resulting in a total of more than 150 distinct measurements in 10 different lineages (with each lineage containing more than 15 cells). For each lineage, mean and CV of GFP concentration were computed. The mean and standard deviation of these values over all lineages were used as estimates of the means, CVs and respective error-bars (Fig J).

S1.1.13 Information on the growth conditions.

Table C. Description of the 8 environmental conditions in which the library of native *E.coli* promoters [1] has been grown. The chosen environmental conditions comprised a MOPS based synthetic rich media, minimal media (M9) with different carbon sources (glucose, lactose and glycerol), an osmotic and DNA damage stress (0.4M NaCl and Ciprofloxacin 1.5 ng/ml both supplemented with glucose), as well as two time points in stationary phase (16 and 30 hours of growth in M9 glucose).

Condition	Condition during overnights	Hours of growth before FACS measurements	Dilution after ON	Growth rate +/- sd (h^{-1})	Mean area +/- sd (in μm^2)
Synthetic Rich*	same	2h	1/1000	1.59 +/- 0.28	4.3 +/- 1.4
M9** + Ciprofloxacin 1.5 ng/ml (+ 0.2% glucose)	M9 + 0.2% glucose	4h	1/200	0.69 +/- 0.07	2.7 +/- 1
M9** + 0.2% glucose	same	4h	1/200	0.67 +/- 0.05	2+/- 0.6
M9** + 0.2% lactose	same	4h	1/200	0.58 +/- 0.03	2.1+/- 0.6
M9** + 0.2% glycerol	same	5h	1/200	0.5 +/- 0.11	1.6 +/- 0.5
M9** + 0.4M NaCl (+ 0.2% glucose)	same	15h	1/500	0.37 +/- 0.03	1.4 +/- 0.3
Stationary phase 16h (M9** 0.2% glucose)	same	16h	1/200	x	1.3 +/- 0.4
Stationary phase 30h (M9** 0.2% glucose)	same	30h	1/200	x	x

* MOPS based. Commercially available (Teknova M2105)

** Prepared as follows: 0.1mM $CaCl_2$, 1mM $MgSo_4$, 1 x M9 salts (Sigma M6030), 50 µg/ml Kanamycin, *d*H20

S1.2 Supplementary Figures

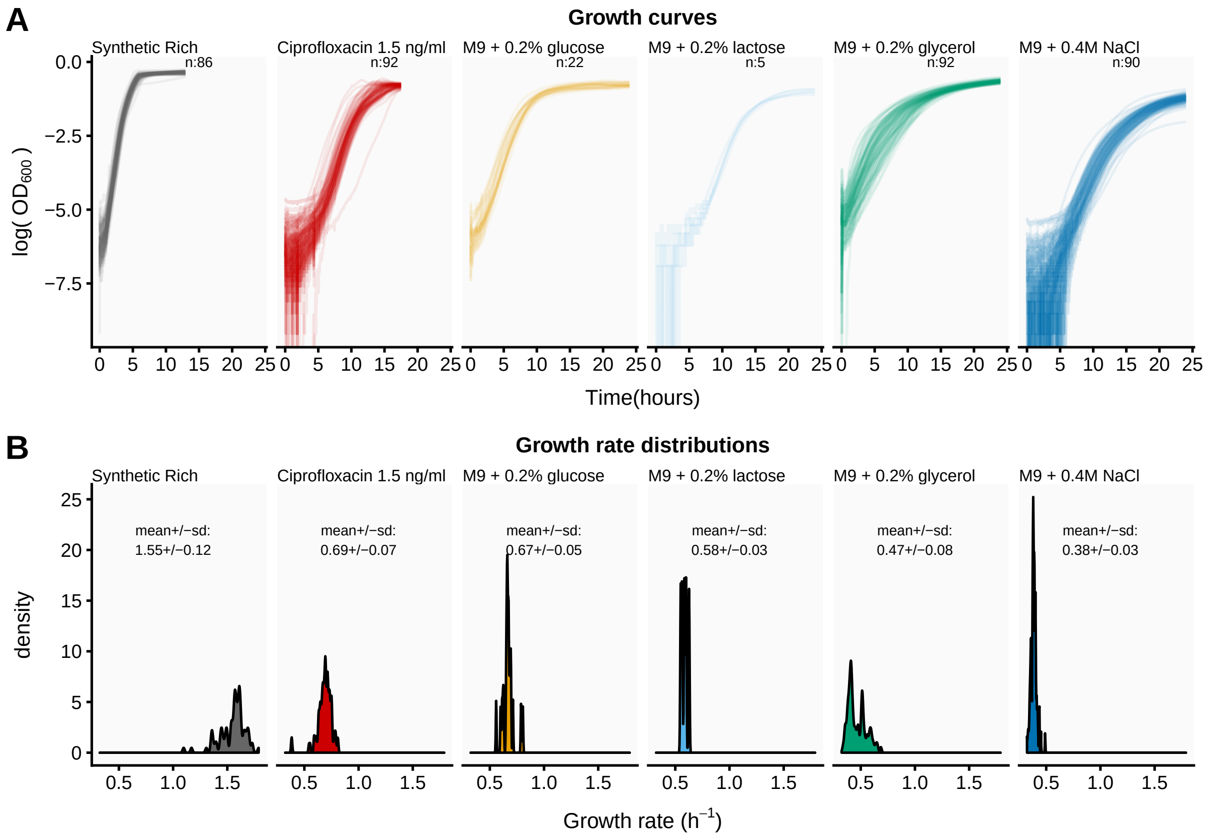

Fig A. Growth curves across conditions. **(A)** OD_{600} (log-scale, y-axis) as a function of time (in hours, x-axis). We measured OD_{600} in individual strains growing in bulk at intervals of 90 seconds during 15 to 25 hours. The number of strains used per condition is indicated in each panel. **(B)** Density distribution of the estimated growth rates in each condition. The growth-rate α was defined as the slope of a linear fit of $\log(OD_{600})$ against time. The underlying data for Fig. A can be found at `https://doi.org/10.5281/zenodo.4662163`.

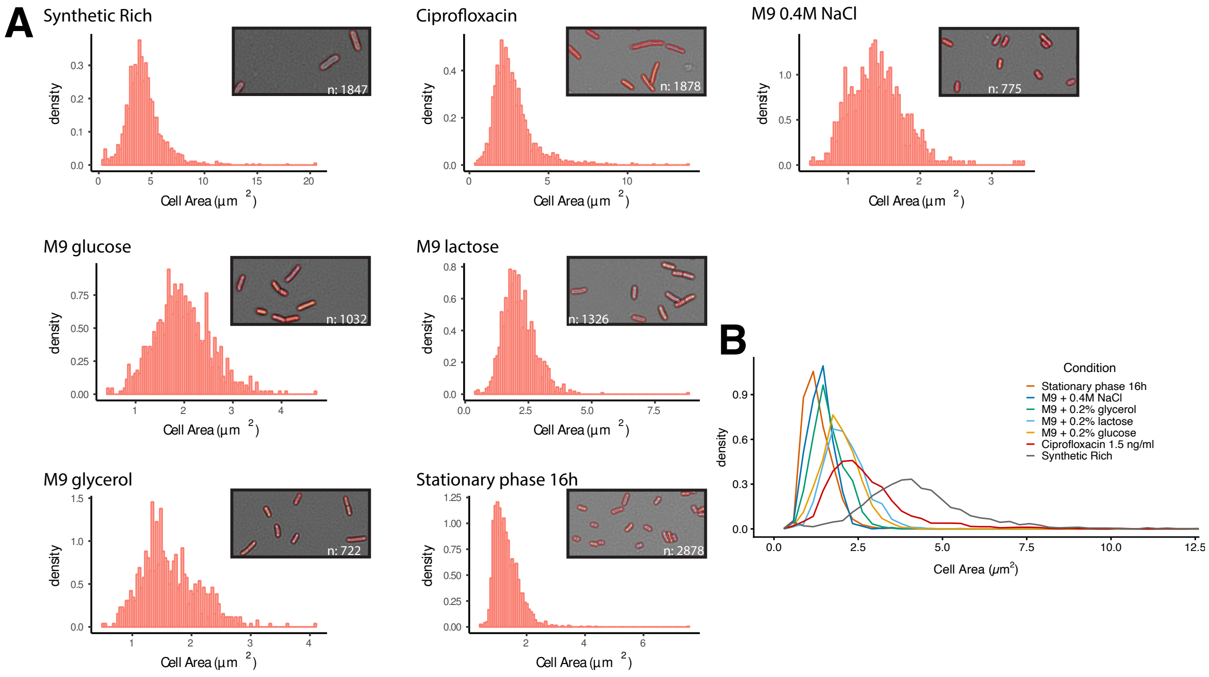

Fig B. Cell sizes distributions. (A) Histograms of the distribution of single-cell areas (μm^2, x-axis) in each condition. The insets in each condition show segmentation examples together with the number of cells used to estimate the mean and standard-deviation of the areas. **(B)** Kernel-density estimates of the distribution of areas across all conditions (Areas bigger than 12.5 μm^2 are not shown). The underlying data for Fig. B can be found at `https://doi.org/10.5281/zenodo.4662163`.

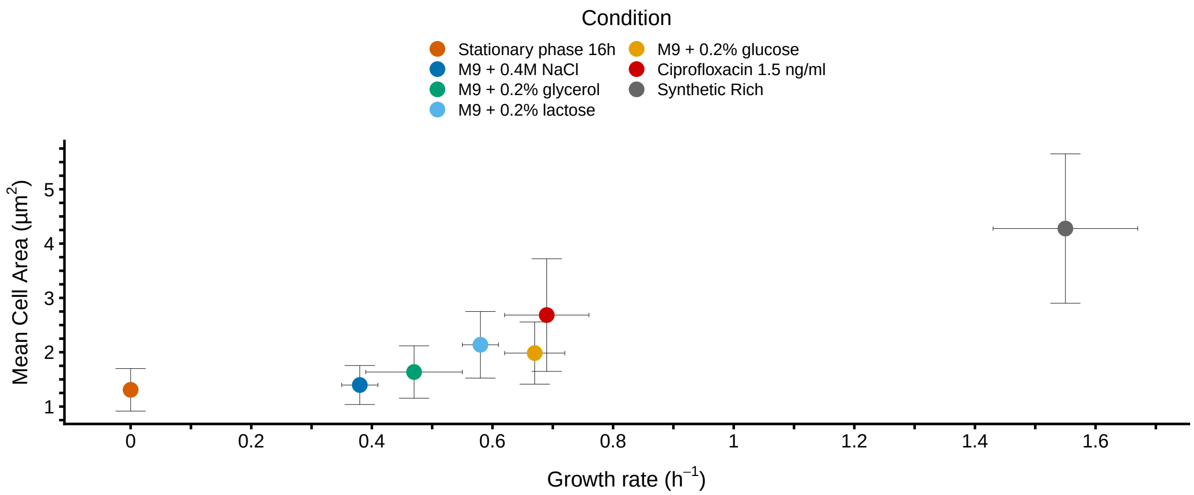

Fig C. Cell area as a function of growth rate. Mean cell area (in μm^2) as a function of the growth rate (h^{-1}) in all conditions except stationary phase 30h. Cross-hairs indicate standard-deviations. The underlying data for Fig. C can be found in S1 data and `https://doi.org/10.5281/zenodo.4662163`.

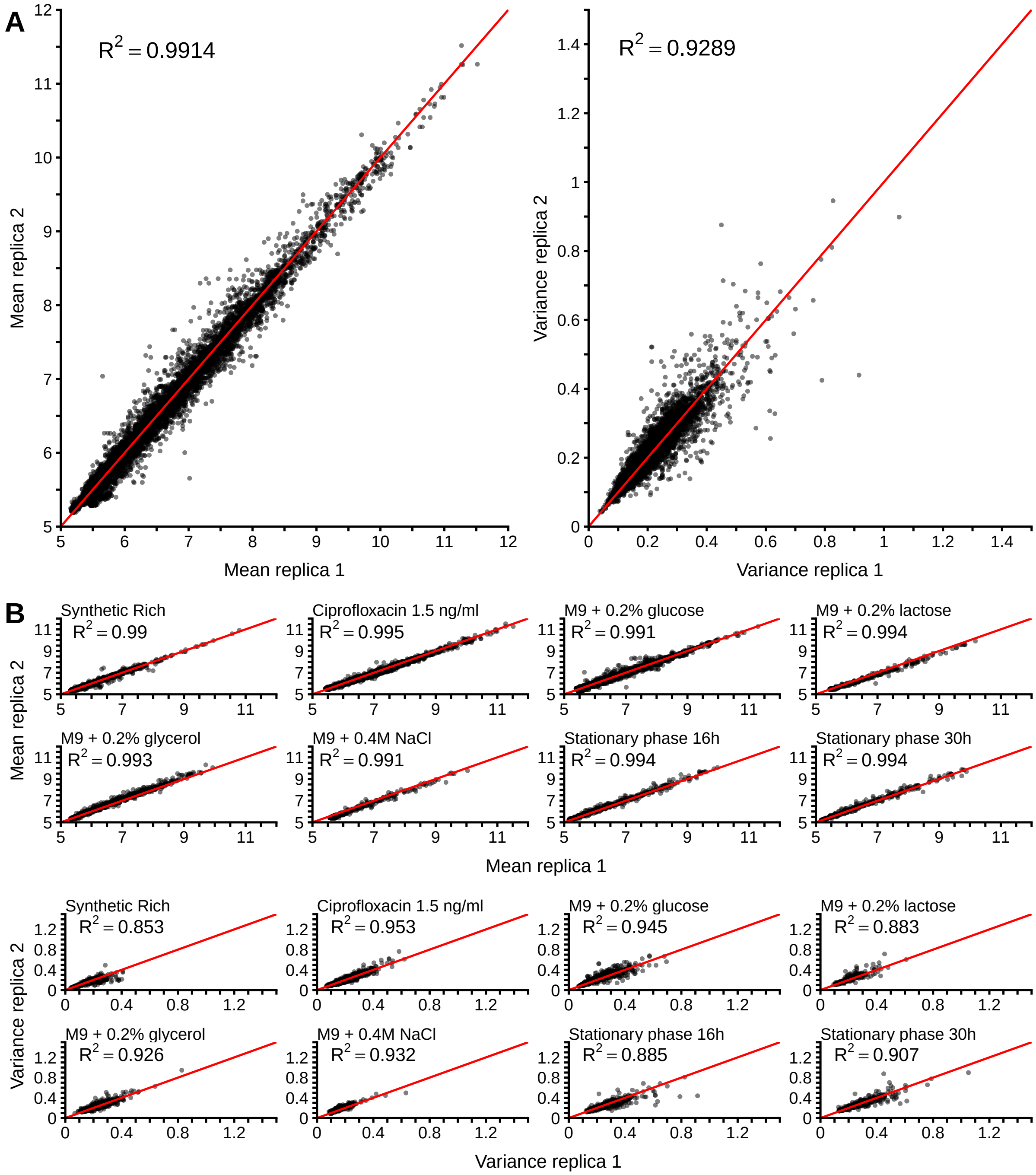

Fig D. Reproducibility of measured means and variances. (A) Means (left-panel) and variances (right-panel) of promoters (each represented by a black dot) measured on different days. The Pearson squared-correlations are indicated in each panel. **(B)** Reproducibility of means (top panel) and variances (bottom panel) separately for each condition. Pearson squared correlations are indicated in each panel. The underlying data for Fig. D can be found in S1 data and `https://doi.org/10.5281/zenodo.4662163`.

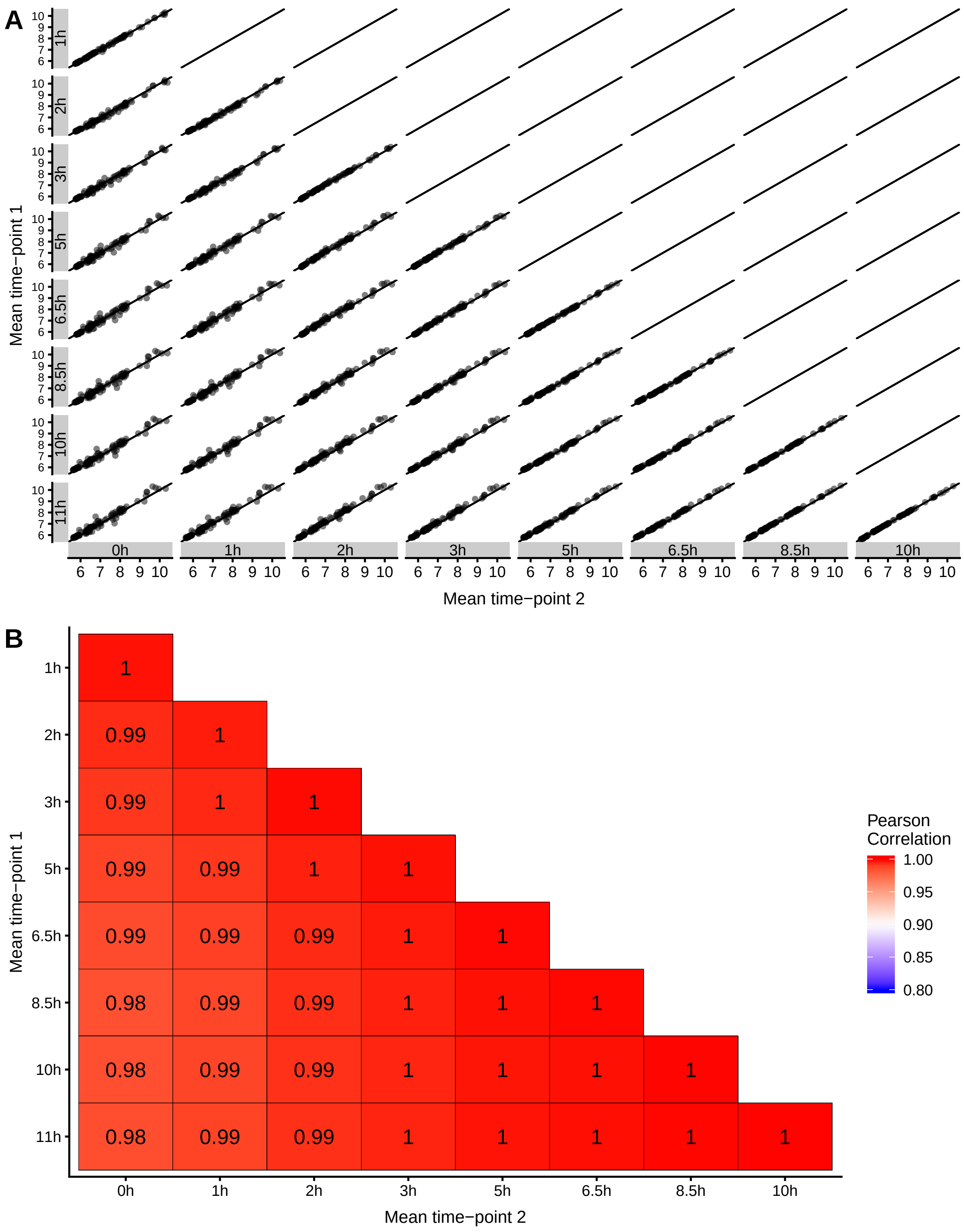

Fig E. Reproducibility of measured mean fluorescences at different time-points during growth. **(A)** Correlations of mean expression levels for 95 promoters from the library, measured at consecutive time points during growth in M9 + 0.4M NaCl (+ 0.2% glucose). The time points range between 0h (freshly diluted culture) and 11 hours. The grey boxes on the axes indicate the time points that are being compared. **(B)** R^2 Pearson correlation coefficients of measured mean expression levels for all pairs of timepoints. The underlying data for Fig. E can be found at `https://doi.org/10.5281/zenodo.4662163`.

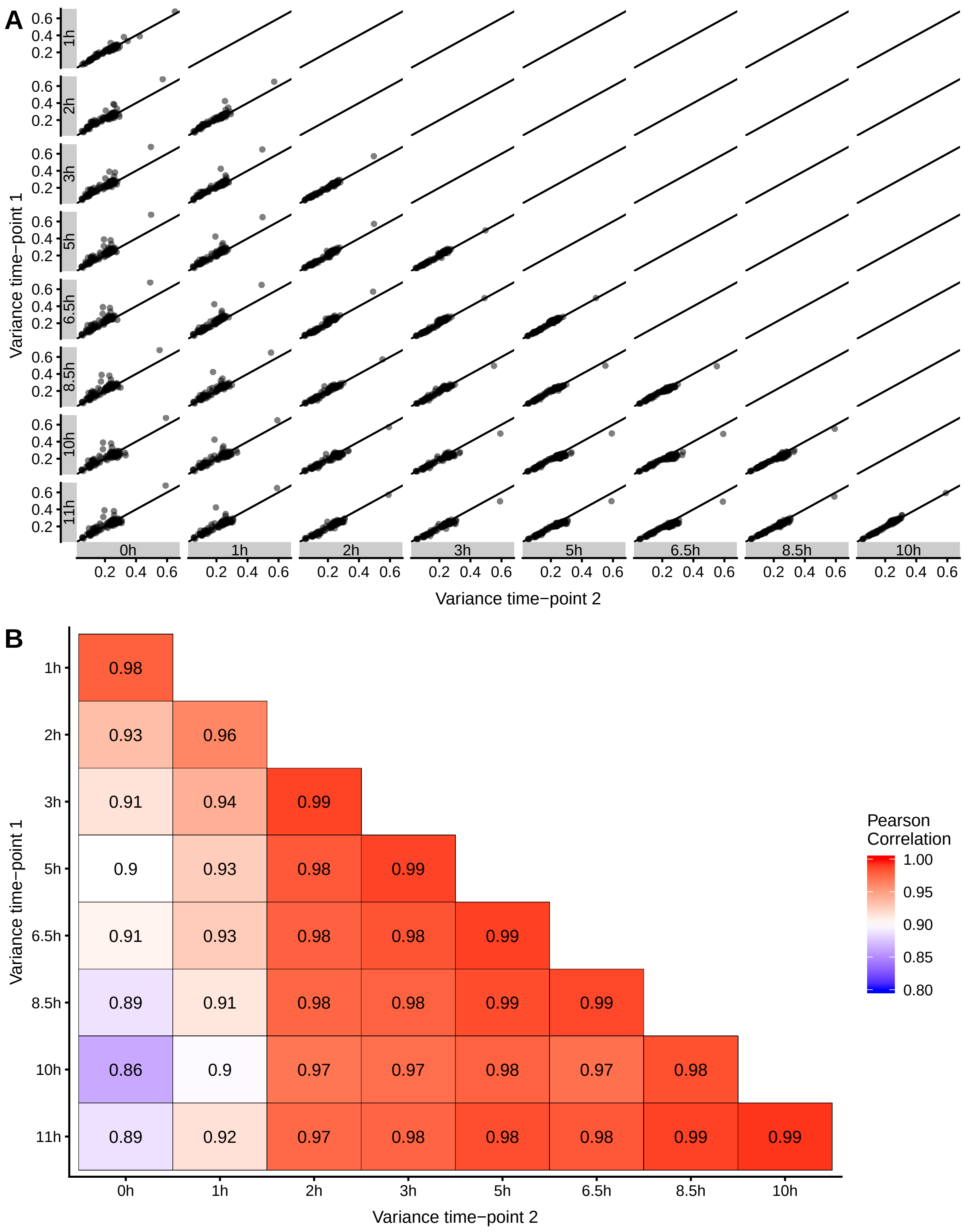

Fig F. Reproducibility of measured fluorescence variances at different time-points during growth. (A) Correlations of variance in expression levels for 95 promoters from the library, measured at consecutive time points during growth in M9 + 0.4M NaCl (+ 0.2% glucose). The time points range between 0h (freshly diluted culture) and 11 hours. The grey boxes on the axes indicate the time points that are being compared. **(B)** R^2 Pearson correlation coefficients of measured variances in expression levels for all pairs of timepoints. The underlying data for Fig. F can be found at `https://doi.org/10.5281/zenodo.4662163`.

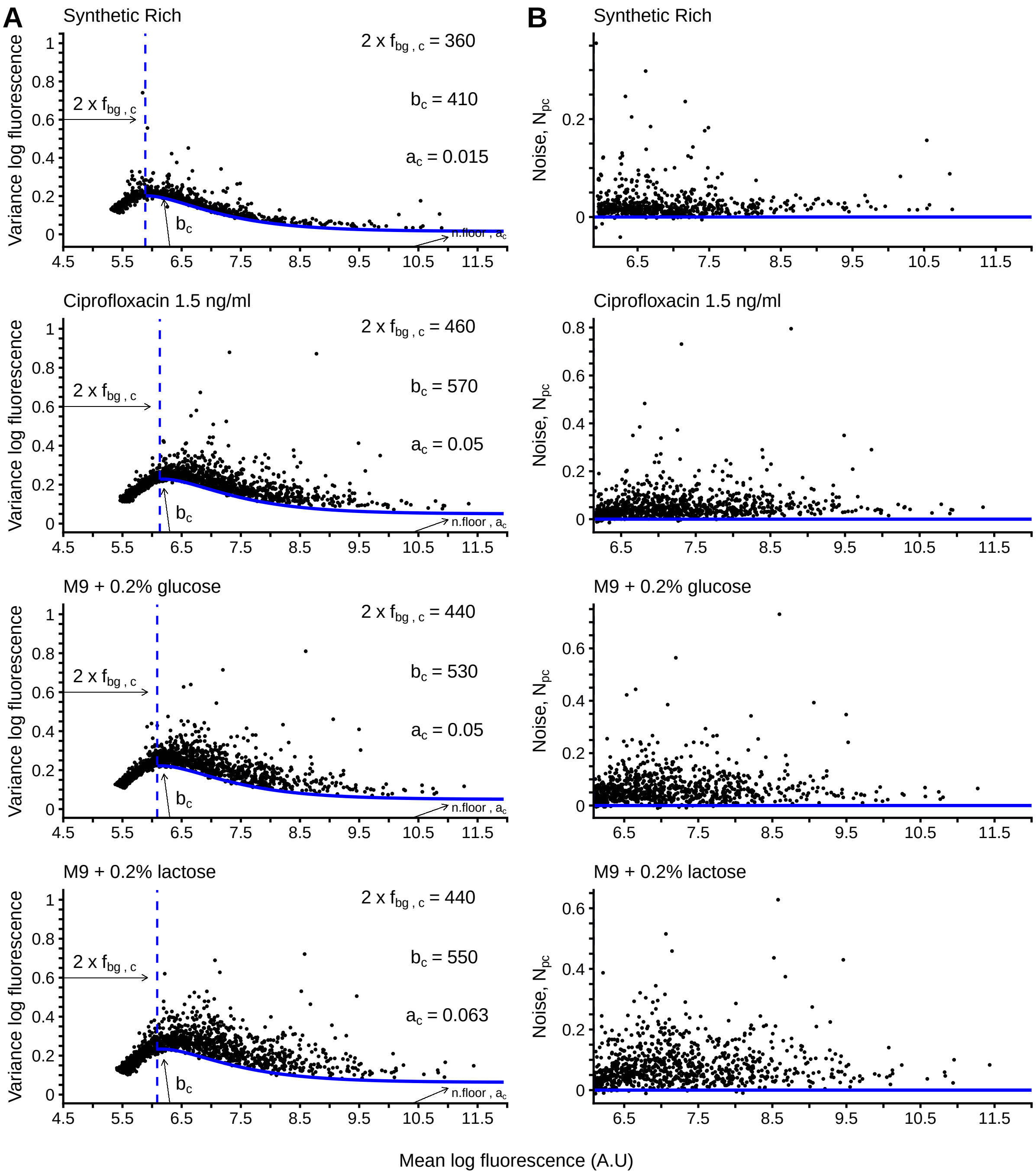

Fig G. Means and variances of promoters from the library in all conditions. (A) Variance as a function of mean for all promoters measured in each condition. Each promoter is represented by a black dot. The blue line indicates the predicted minimal variance as a function of mean. Thia model breaks down for fluorescence levels close to background (left of the vertical blue dashed line), thus we only considered promoters above it. The number of promoters measured per condition is annotated inside each panel. **(B)** Noise-level N_{pc} as a function of mean after correcting for the mean-dependent noise floor, i.e. differences between measured variance and minimal variance (Figure continued on next page). The underlying data for Fig. G can be found at `https://doi.org/10.5281/zenodo.4662163`.

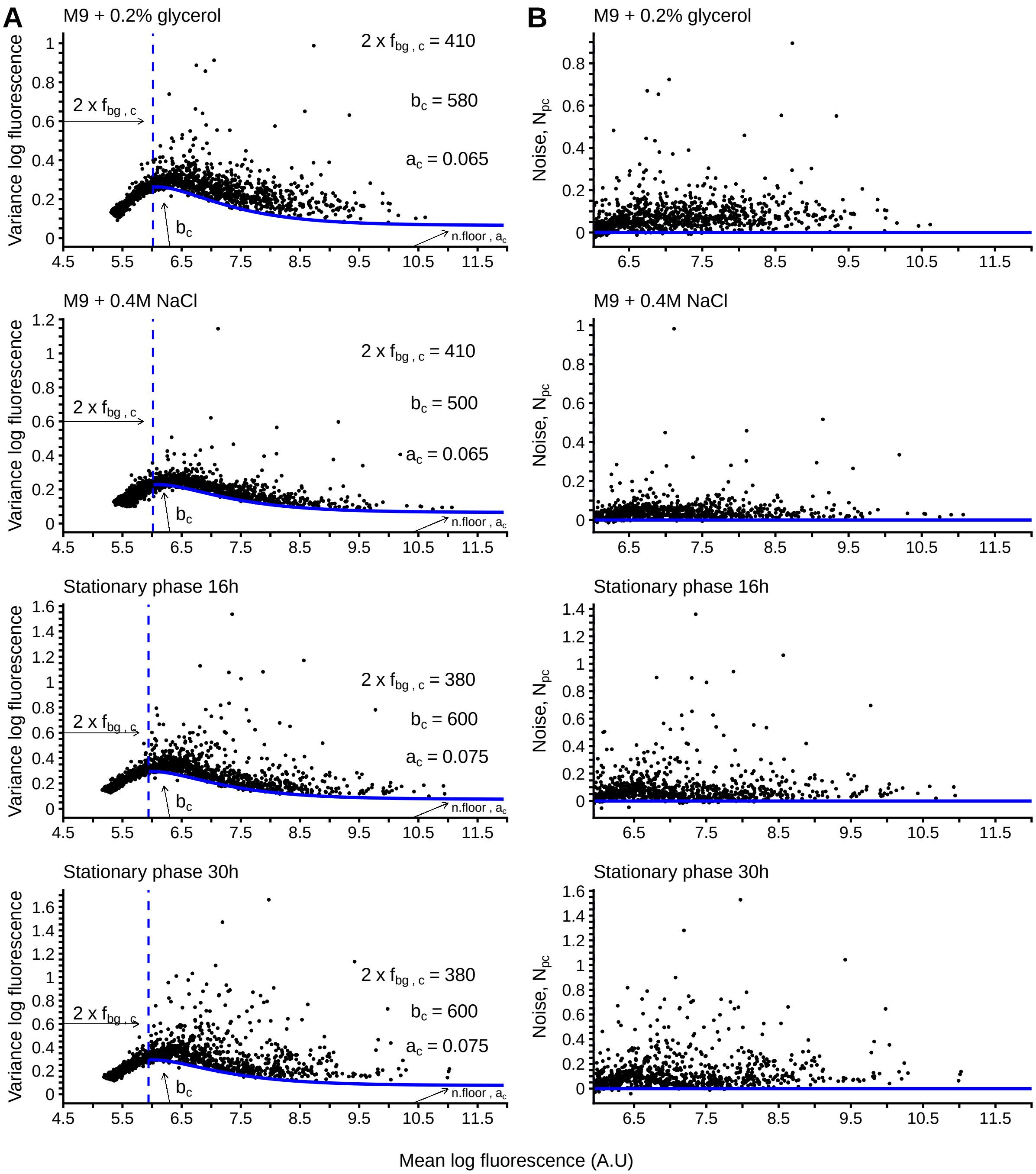

Fig G. Means and variances of promoters from the library in all conditions. (A) Variance as a function of mean for all promoters measured in each condition. Each promoter is represented by a black dot. The blue line indicates the predicted minimal variance as a function of mean. This model breaks down for fluorescence levels close to background (left of the vertical blue dashed line), thus we only considered promoters above it. The number of promoters measured per condition is annotated inside each panel. **(B)** Noise-level N_{pc} as a function of mean after correcting for the mean-dependent noise floor, i.e. differences between measured variance and minimal variance. The underlying data for Fig. G can be found at `https://doi.org/10.5281/zenodo.4662163`.

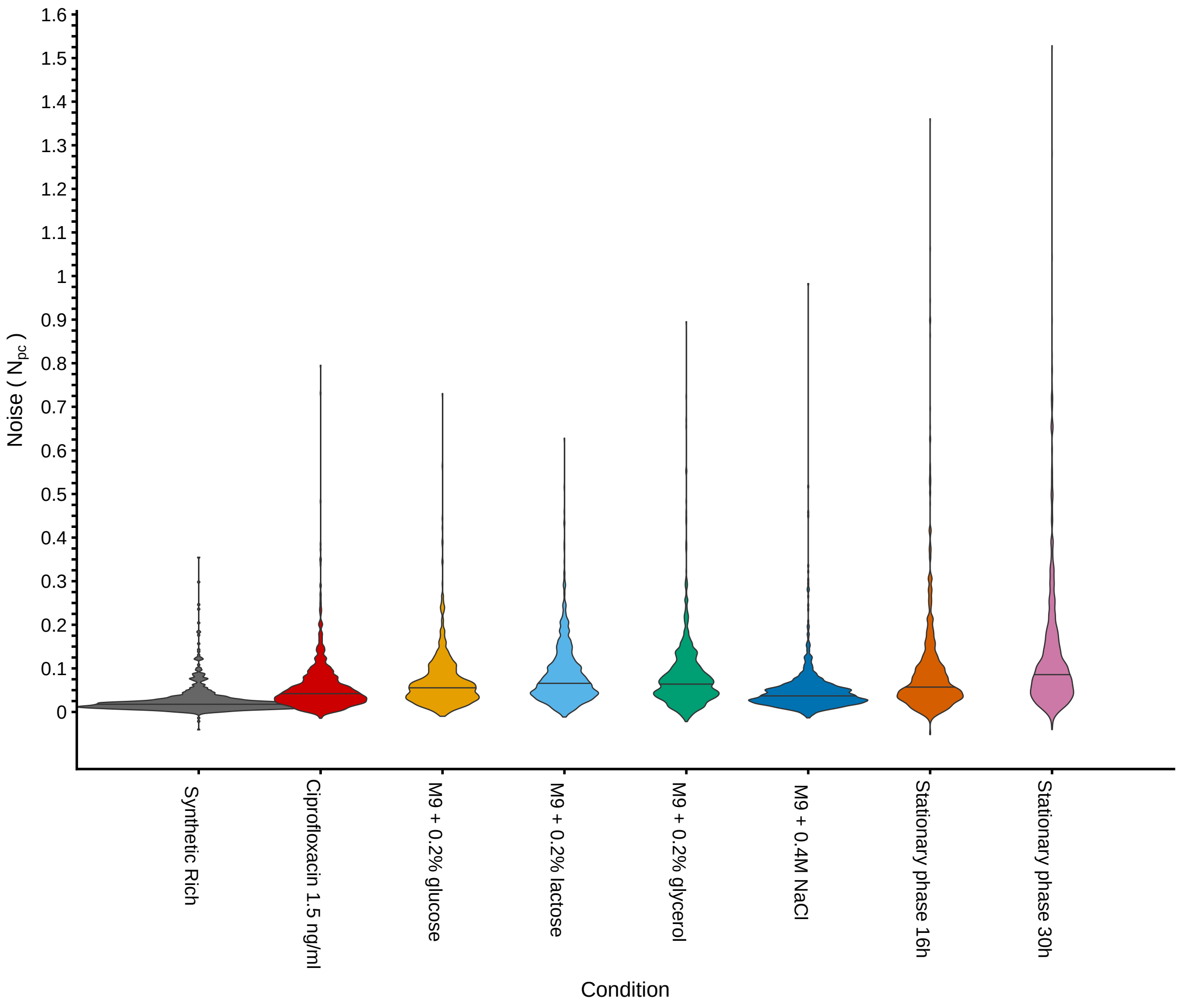

Fig H. Noise levels across all conditions. The violin plots show the distributions of noise levels for all promoters in each of the measured conditions (i.e. the same data as Fig. 1E). The horizontal lines indicate the medians. The underlying data for Fig. H can be found at `https://doi.org/10.5281/zenodo.4662163`.

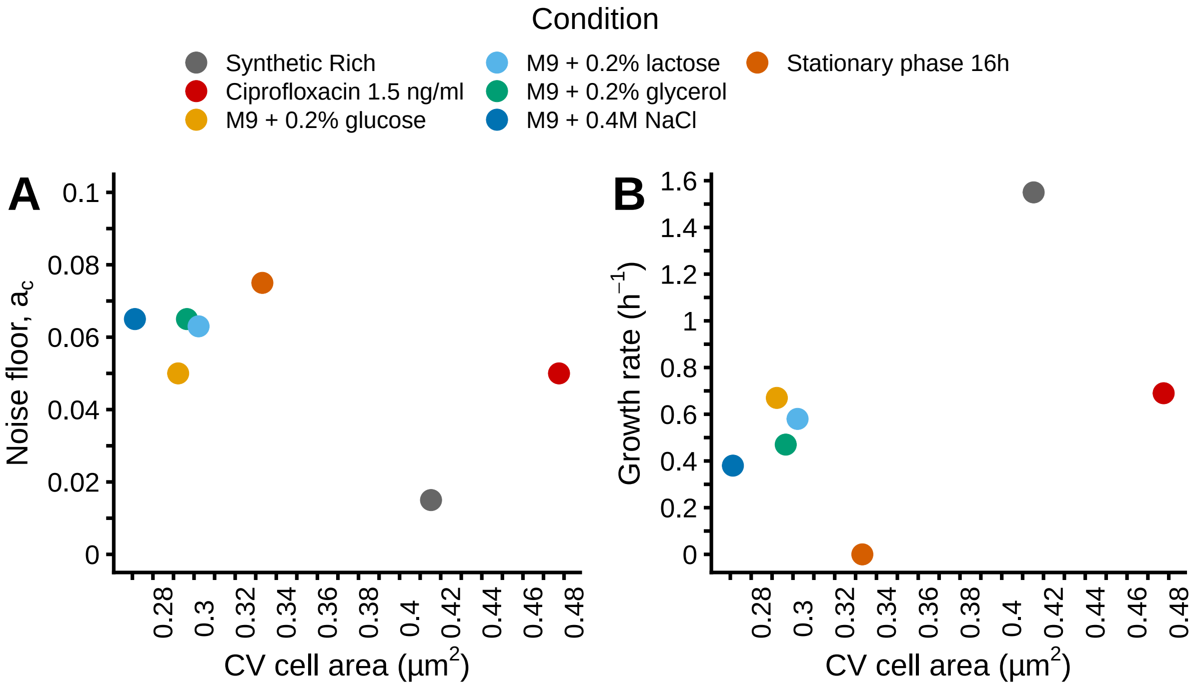

Fig I. Single-cell variability in cell size versus the noise floor and growth rate. We estimated the coefficient of variation (CV) in cell size (distributions shown in Fig C) in our measured conditions (*stationary phase at 30h not shown*) to investigate if the noise floor is also affected by fluctuations in cell size, as well as the relationship between the CV in cell size and growth rate. **(A)** The noise floor as a function of the CV in cell size (area in μm^2) in the respective condition. **(B)** The growth rate as a function of the CV in cell size in each respective condition. The underlying data for Fig. I can be found in S1 data and `https://doi.org/10.5281/zenodo.4662163`.

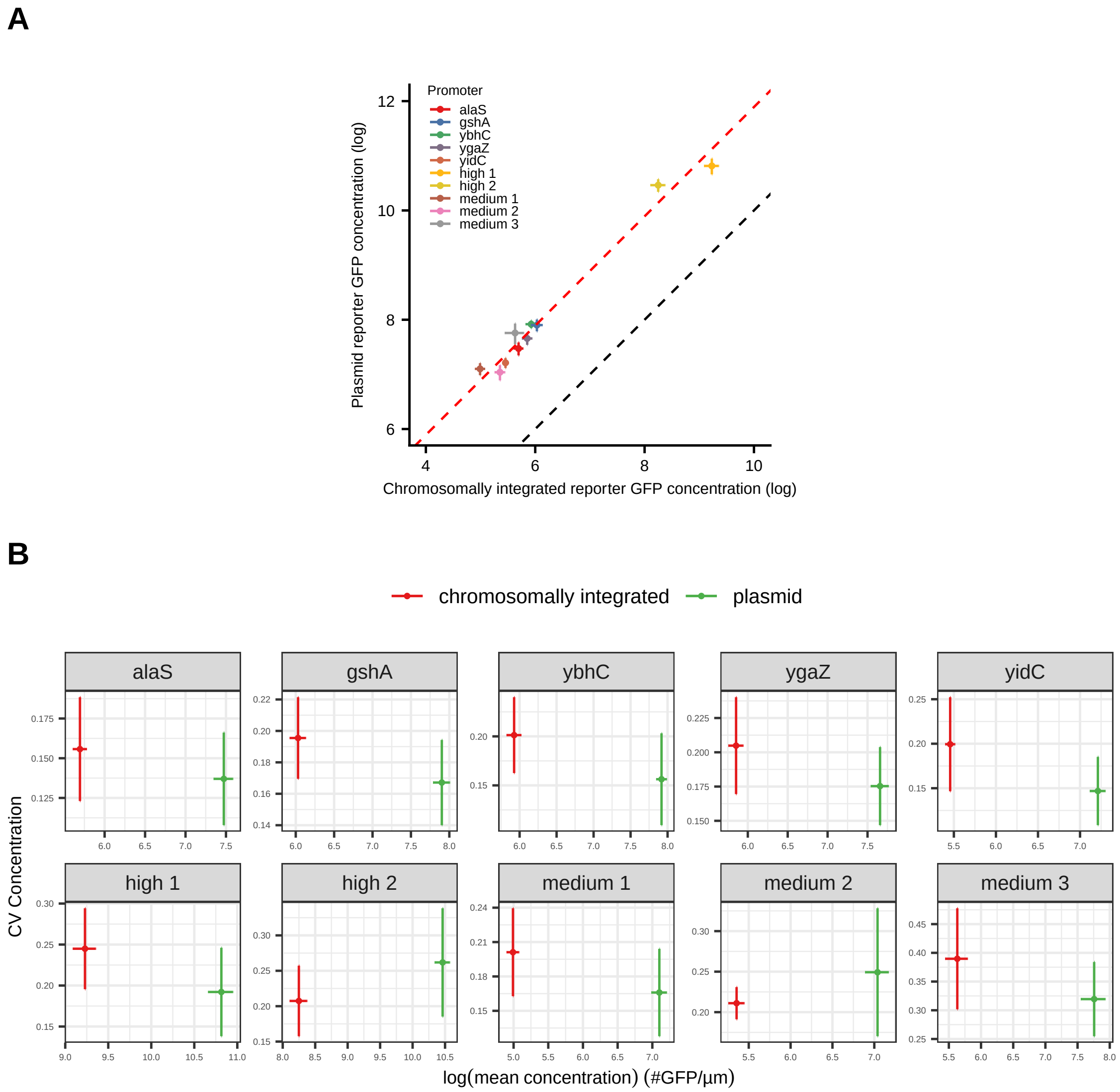

Fig J. Means and coefficients of variation of corresponding plasmid-based and chromosomally integrated reporters. We integrated 5 native promoters (regulating *alaS, gshA, ybhC, ygaZ, yidC*) and 4 synthetic promoters (high 1, high 2, medium 1, medium 2) showing noise levels close to the noise floor into the same location in *E. coli*'s MG1655 chromosome. In addition, we integrated 1 synthetic promoter characterized by higher expression noise (medium 3). For each pair of corresponding reporter constructs (plasmid-based and chromosomally integrated), the mean GFP concentration and CV in concentration were measured across single cells using fluorescence microscopy (see SI Methods and Texts). **(A)** Comparison of the log of the mean concentration of the plasmid vs chromosomal reporters. Black dashed line: y=x. Red dashed line: linear regression with slope 1 (inferred intercept is 1.89). **(B)** CV of concentrations as a function of the log of the mean concentration for plasmid (green) and chromosomal (red) reporters. Error bars (**(A)** and **(B)**) are standard deviations across independent lineages ($n >= 10$). Note that CV for plasmid-based reporters is not systematically higher or lower than those of the chromosomally integrated reporters. The underlying data for Fig. J can be found in S1 data.

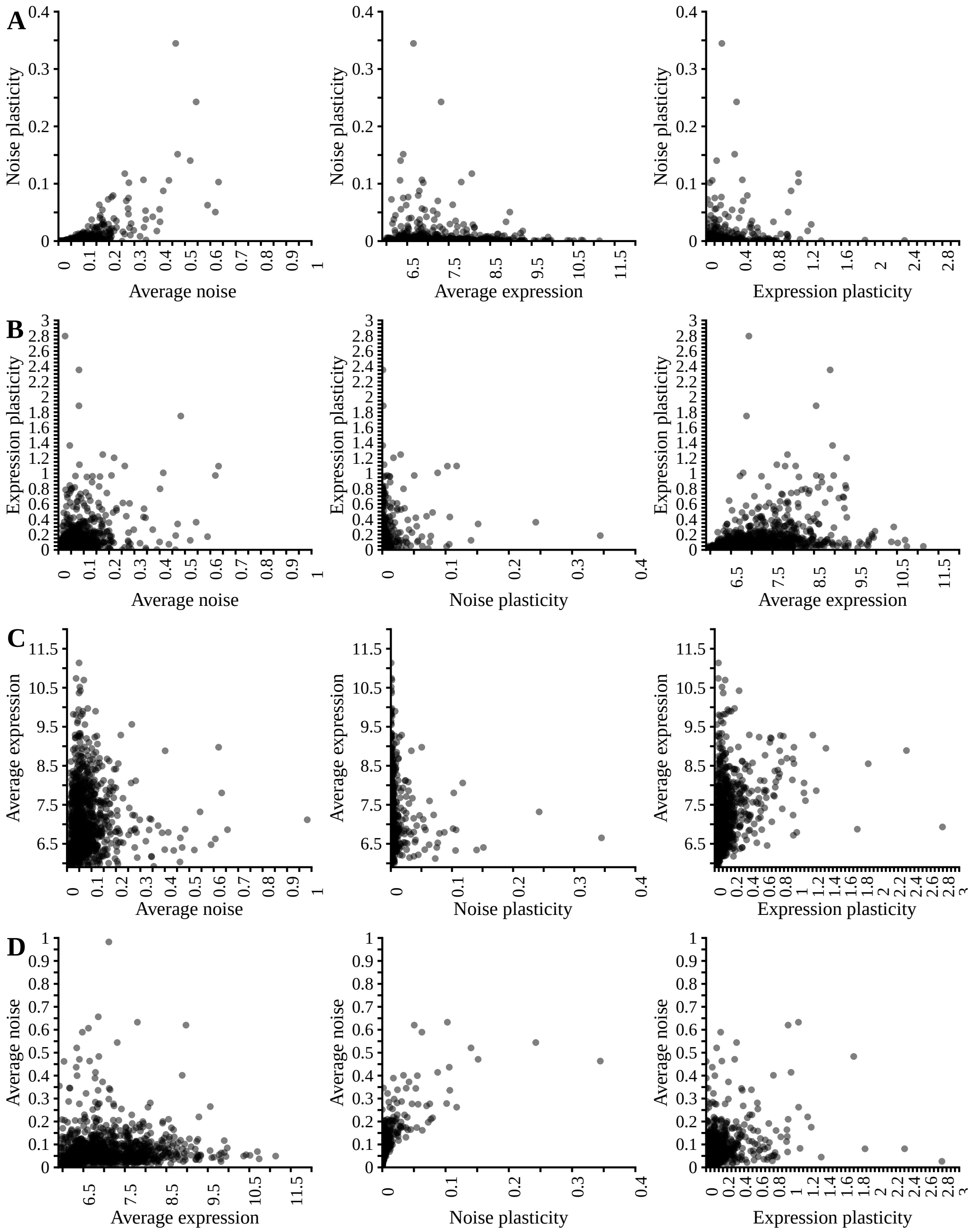

Fig K. Relationship between noise, expression plasticity, and average noise and expression. **(A)** Each subplot shows the plasticity in noise (variance in noise across all conditions) across all measured conditions as a function of the average noise, average expression and expression plasticity for each promoter. **(B)** Expression plasticity (variance in expression across all conditions) as a function of average noise, noise plasticity and average expression. **(C)** Average expression as a function of average noise, noise plasticity and expression plasticity. **(D)** Average noise as a function of average expression, noise plasticity and expression plasticity. Each black dot represents one promoter of the library. The underlying data for Fig. K can be found in S1 data and `https://doi.org/10.5281/zenodo.4662163`.

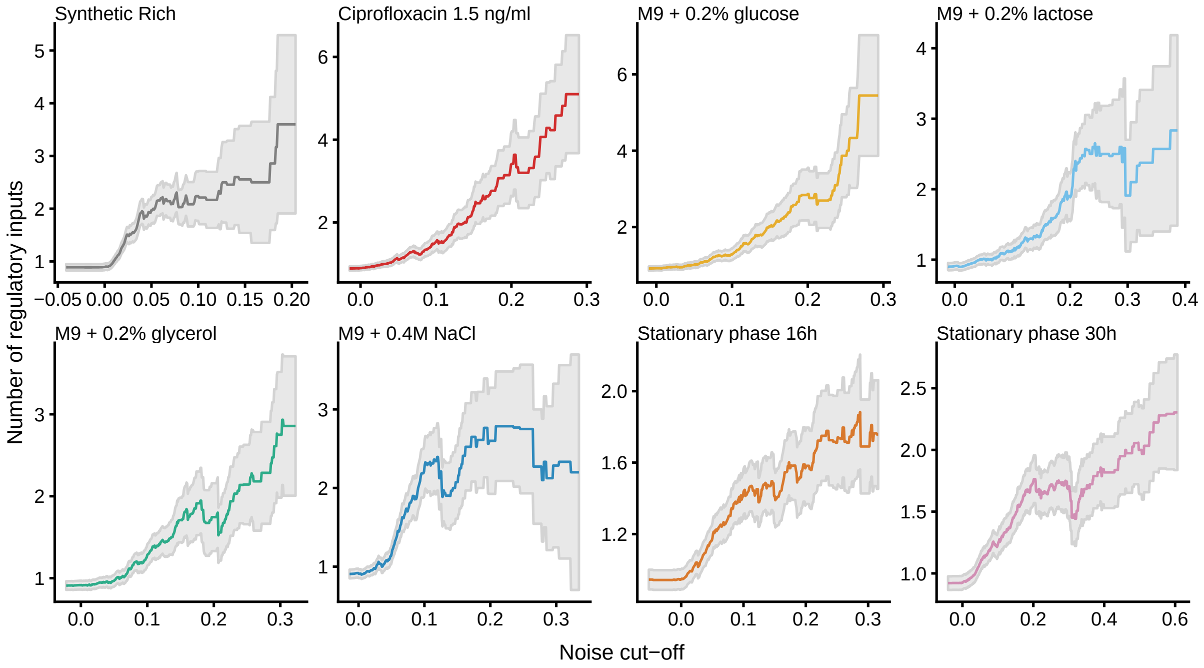

Fig L. Noise as a function of number of regulatory inputs. In each condition we sorted promoters by their noise level N_{pc}, and calculated the mean and standard-error of the number of known regulatory inputs (y-axis) of all promoters above a cut-off in N_{pc} (x-axis). Regulatory input annotations were taken from RegulonDB [4]. The underlying data for Fig. L can be found in S1 data.

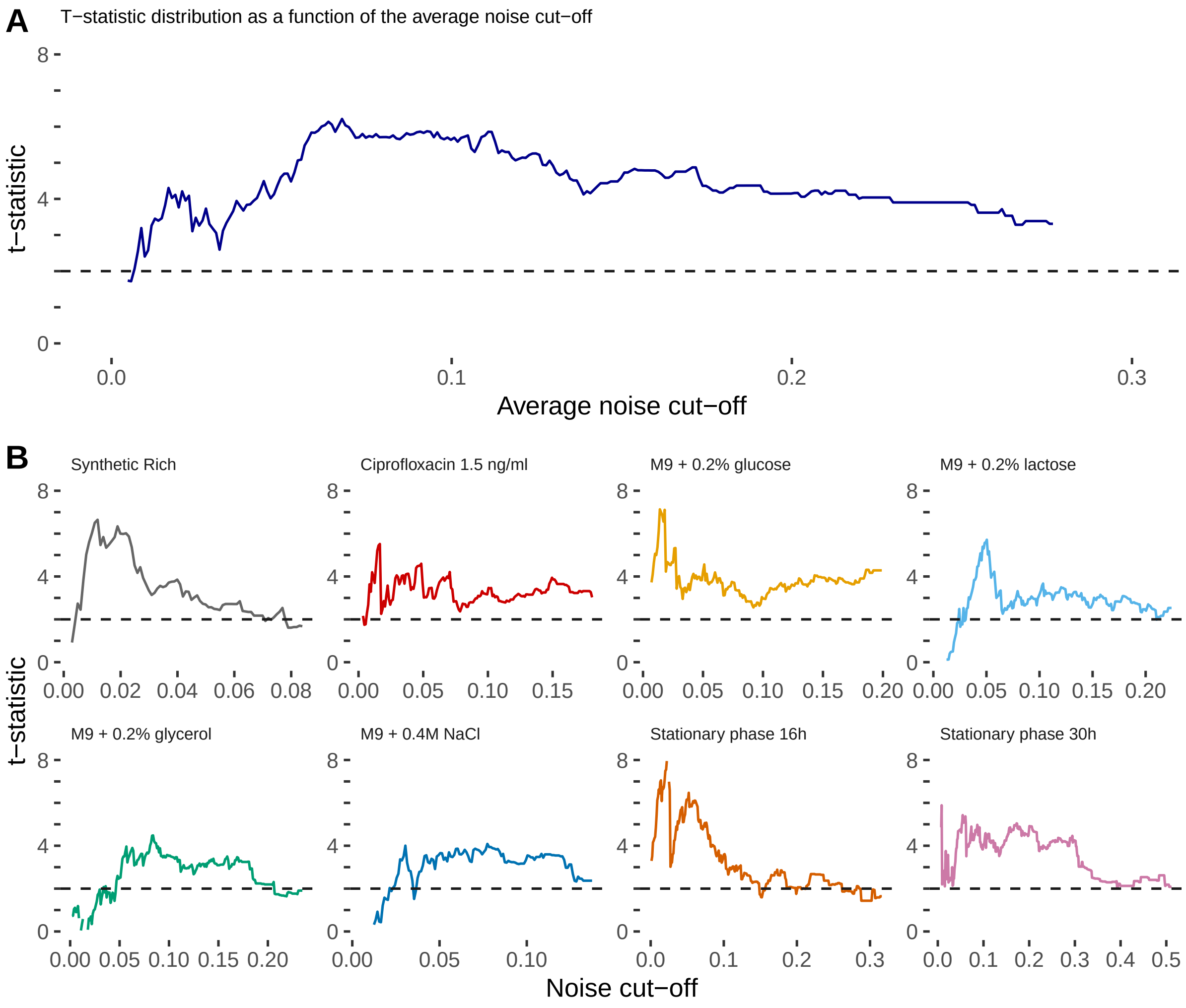

Fig M. Significance of the relationship between high noise and regulatory inputs (S1 data). t-statistics (y-axis, see SI Methods and Texts) of the difference in mean number of regulatory inputs between promoters with noise above and below a given cut-off in noise level (x-axis). **(A)** The t-statistic as a function of an average noise across all conditions. **(B)** The t-statistic for each of our measured conditions separately (condition name written at the top of each panel). The dashed lines correspond to a t-statistics of 2, corresponding roughly to two standard-deviations. The underlying data for Fig. M can be found in S1 data.

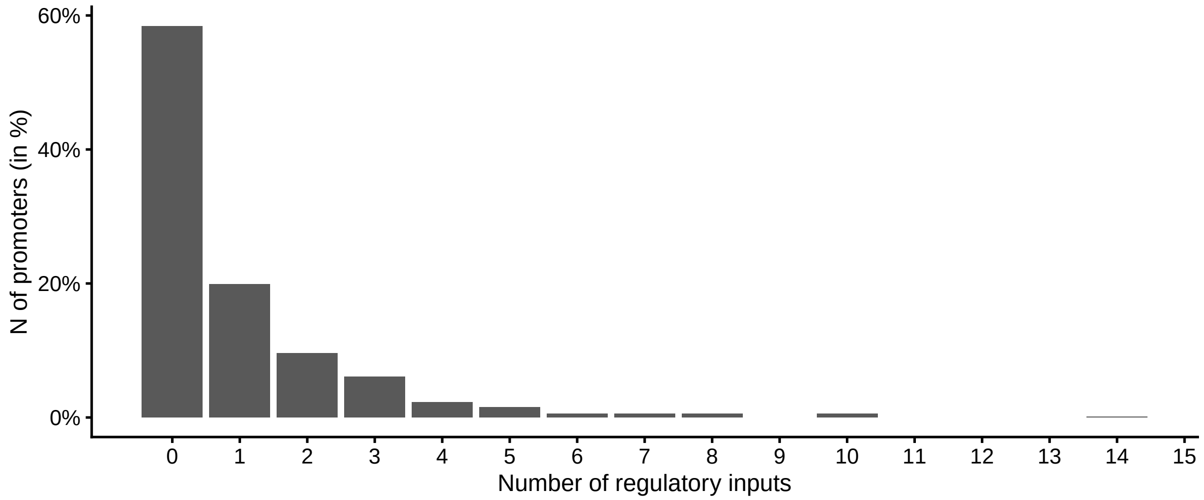

Fig N. Percentage of promoters with annotated regulatory inputs. Histogram of the percentage of promoters (y-axis) with a given number of annotated regulatory inputs (x-axis) as documented in RegulonDB [4]. Note that no regulatory input is known for almost 60% of promoters and that promoters with 5 or more regulatory inputs are very rare. The underlying data for Fig. N can be found in S1 data and `https://doi.org/10.5281/zenodo.4662163`.

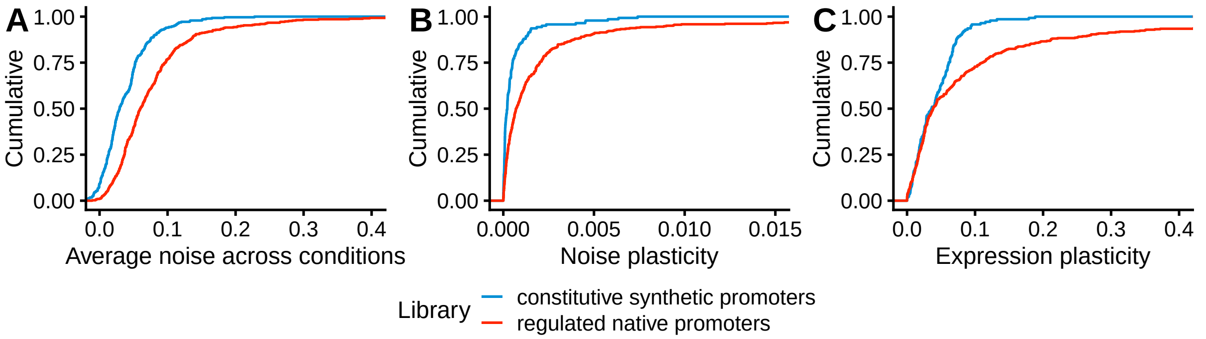

Fig O. Constitutive promoters show lower average noise, noise plasticity, and expression plasticity than regulated promoters. Cumulative distributions of the average expression noise (**A**), noise plasticity (**B**), and expression plasticity (**C**) across four conditions (M9 + 0.2% glucose, M9 + 0.2% glycerol, M9 + 0.2% lactose, M9 + 0.4M NaCl) for synthetic constitutive promoters (blue line, see Methods) and regulated native promoters (promoters with at least 1 annotated regulatory site beyond the sigma site according to RegulonDB [4], red line). The underlying data for Fig. O can be found in S1 data and `https://doi.org/10.5281/zenodo.4662163`.

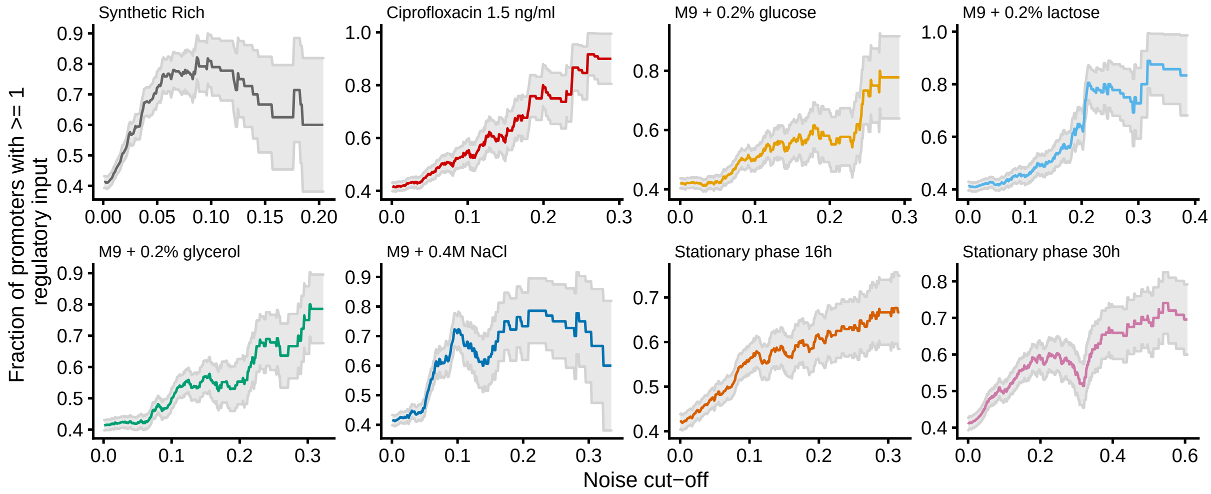

Fig P. Fraction of promoters with at least 1 annotated regulatory input as a function of noise. In each condition, we sorted promoters by their noise level N_{pc}, and estimated the fraction and standard error (grey area) of promoters with at least 1 annotated regulatory input (y-axis) above a cut-off in noise N_{pc}, as a function of noise (x-axis). Note that, across conditions, $70-90\%$ of high noise promoters have at least one known regulatory input. The underlying data for Fig. P can be found in S1 data and `https://doi.org/10.5281/zenodo.4662163`.

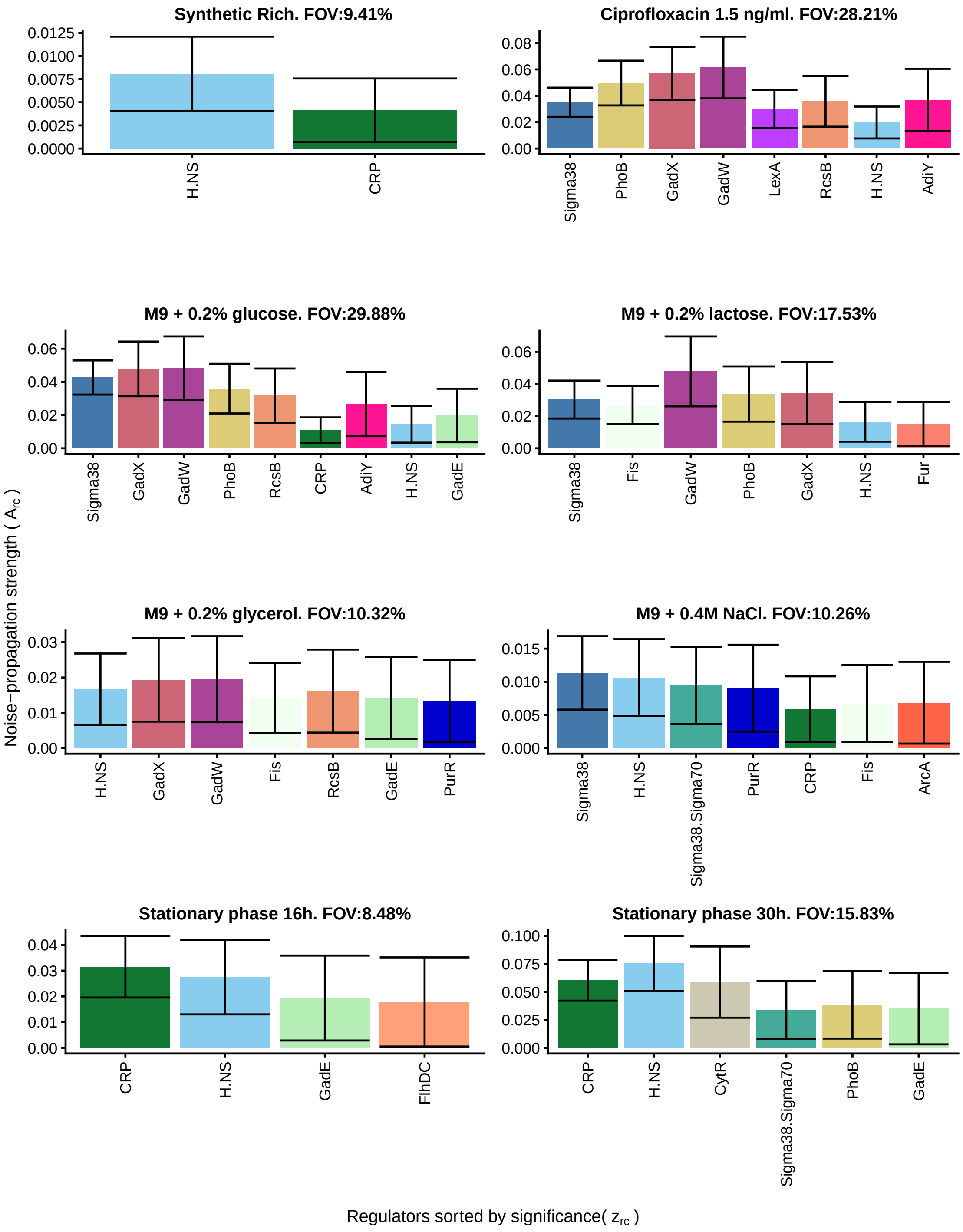

Fig Q. Strongest noise-propagators in each condition. Each panel corresponds to one growth condition and shows the inferred noise propagation strengths A_{rc} for the transcription factors for which $A_{rc} > \delta A_{rc}$ in that condition. The TFs are sorted by their overall significance z_r. The condition is indicated above each panel together with the fraction of variance (FOV) explained by the model. The underlying data for Fig. Q can be found in S1 data and `https://doi.org/10.5281/zenodo.4662163`.

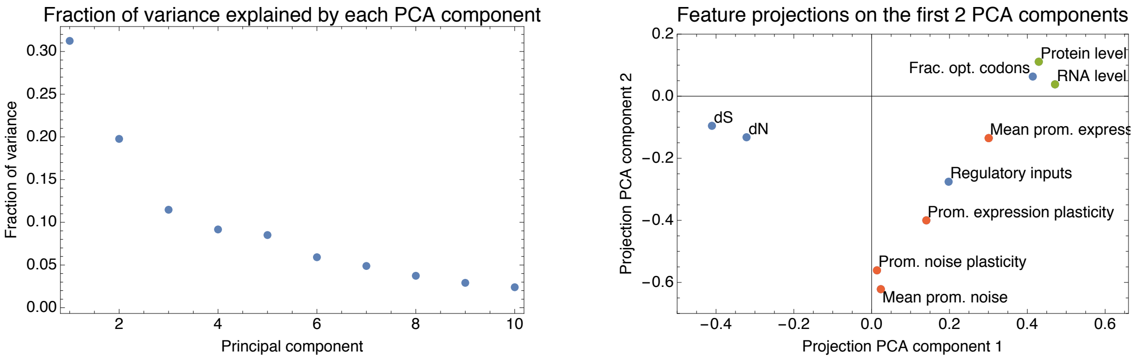

Fig R. Principal component analysis of the 10 **gene features. (A)** Fractions of the total variance in gene features captured by each of the PCA components. Note that the first two components together capture more than 50% of the variance. **(B)** Projection of each of the 10 features on the first two PCA components. Expression levels from the literature are shown in green, sequence features are shown in blue, and gene expression features measured in this study are shown in red. The underlying data for Fig. R can be found in S1 data.

References

1. Zaslaver A, Bren A, Ronen M, Itzkovitz S, Kikoin I, Shavit S, et al. A comprehensive library of fluorescent transcriptional reporters for Escherichia coli. Nature Methods. 2006 8;3(8):623–628. Available from: http://www.nature.com/doifinder/10.1038/nmeth895.

2. Galbusera L, Bellement-Theroue G, Urchueguia A, Julou T, van Nimwegen E. Using fluorescence flow cytometry data for single-cell gene expression analysis in bacteria. PLoS One. 2020;15(10):e0240233. Available from: https://doi.org/10.1371/journal.pone.0240233.

3. Wolf L, Silander OK, van Nimwegen E. Expression noise facilitates the evolution of gene regulation. eLife. 2015 6;4:e05856. Available from: https://doi.org/10.7554/eLife.05856.

4. Santos-Zavaleta A, Salgado H, Gama-Castro S, Sánchez-Pérez M, Gómez-Romero L, Ledezma-Tejeida D, et al. RegulonDB v 10.5: tackling challenges to unify classic and high throughput knowledge of gene regulation in E. coli K-12. Nucleic Acids Research. 2019 1;47(D1):D212–D220. Available from: https://academic.oup.com/nar/article/47/D1/D212/5160972.

5. Suzuki H, Forrest AR, van Nimwegen E, Daub CO, Balwierz PJ, Irvine KM, et al. The transcriptional network that controls growth arrest and differentiation in a human myeloid leukemia cell line. Nature genetics. 2009 5;41(5):553–562. Available from: https://doi.org/10.1038/ng.375.

6. Balwierz PJ, Pachkov M, Arnold P, Gruber AJ, Zavolan M, Van Nimwegen E. ISMARA: automated modeling of genomic signals as a democracy of regulatory motifs. Genome Research. 2014 5;24(5):869–884. Available from: http://www.genome.org/cgi/doi/10.1101/gr.169508.113.

7. Salmon KA, Hung SP, Steffen NR, Krupp R, Baldi P, Hatfield GW, et al. Global gene expression profiling in Escherichia coli K12: Effects of oxygen availability and ArcA. Journal of Biological Chemistry. 2005 4;280(15):15084–15096. Available from: http://www.ncbi.nlm.nih.gov/pubmed/15699038.

8. Arense P, Bernal V, Iborra JL, Cánovas M. Metabolic adaptation of Escherichia coli to long-term exposure to salt stress. Process Biochemistry. 2010 9;45(9):1459–1467. Available from: https://www.sciencedirect.com/science/article/pii/S1359511310002035.

9. Sernova NV, Gelfand MS. Comparative genomics of CytR, an unusual member of the LacI family of transcription factors. PLoS ONE. 2012 9;7(9):e44194. Available from: http://dx.plos.org/10.1371/journal.pone.0044194.

10. Kram KE, Geiger C, Ismail WM, Lee H, Tang H, Foster PL, et al. Adaptation of Escherichia coli to long-term serial passage in complex medium: evidence of parallel evolution. mSystems. 2017 3;2(2). Available from: https://msystems.asm.org/content/2/2/e00192-16.

11. Vassinova N, Kozyrev D. A method for direct cloning of fur-regulated genes: identification of seven new fur-regulated loci in Escherichia coli. Microbiology. 2000 12;146(12):3171–3182. Available from: https://www.microbiologyresearch.org/content/journal/micro/10.1099/00221287-146-12-3171.

12. Dorman CJ. H-NS: a universal regulator for a dynamic genome. Nature Reviews Microbiology. 2004 5;2(5):391–400. Available from: http://www.nature.com/articles/nrmicro883.

13. Tanaka K, Takayanagi Y, Fujita N, Ishihama A, Takahashi H. Heterogeneity of the principal sigma factor in Escherichia coli: the rpoS gene product, sigma 38, is a second principal sigma factor of RNA polymerase in stationary-phase Escherichia coli. Proceedings of the National Academy of Sciences. 1993 4;90(8):3511–3515. Available from: https://doi.org/10.1073/pnas.90.8.3511.

14. Landini P, Egli T, Wolf J, Lacour S. sigmaS, a major player in the response to environmental stresses in Escherichia coli: Role, regulation and mechanisms of promoter recognition. Environmental Microbiology Reports. 2014 2;6(1):1–13. Available from: http://doi.wiley.com/10.1111/1758-2229.12112.

15. Dong T, Schellhorn HE. Control of RpoS in global gene expression of Escherichia coli in minimal media. Molecular Genetics and Genomics. 2009 1;281(1):19–33. Available from: https://doi.org/10.1007/s00438-008-0389-3.

16. Patange O, Schwall C, Jones M, Griffith D, Phillips A, Locke J. Escherichia coli can survive stress by noisy growth modulation. Nature Communications. 2018 12;9(1):5333. Available from: https://doi.org/10.1038/s41467-018-07702-z.

17. You C, Okano H, Hui S, Zhang Z, Kim M, Gunderson CW, et al. Coordination of bacterial proteome with metabolism by cyclic AMP signalling. Nature. 2013 8;500(7462):301–306. Available from: https://doi.org/10.1038/nature12446.

18. Santos-Beneit F. The Pho regulon: a huge regulatory network in bacteria. Frontiers in Microbiology. 2015 4;6. Available from: https://doi.org/10.3389/fmicb.2015.00402.

19. Tucker DL, Tucker N, Ma Z, Foster JW, Miranda RL, Cohen PS, et al. Genes of the GadX-GadW regulon in Escherichia coli. Journal of Bacteriology. 2003 5;185(10):3190–3201. Available from: http://jb.asm.org/cgi/doi/10.1128/JB.185.10.3190-3201.2003.

20. Basan M, Hui S, Okano H, Zhang Z, Shen Y, Williamson JR, et al. Overflow metabolism in Escherichia coli results from efficient proteome allocation. Nature. 2015 12;528(7580):99–104. Available from: http://www.nature.com/doifinder/10.1038/nature15765.

21. Kleman GL, Strohl WR. Acetate metabolism by Escherichia coli in high-cell-density fermentation. Applied and Environmental Microbiology. 1994 11;60(11):3952–3958. Available from: https://www.ncbi.nlm.nih.gov/pmc/articles/PMC201921/pdf/aem00028-0062.

22. Mitosch K, Rieckh G, Bollenbach T. Noisy response to antibiotic stress predicts subsequent single-cell survival in an acidic environment. Cell Systems. 2017 3;4(4):393–403. Available from: http://dx.doi.org/10.1016/j.cels.2017.03.001.

23. Taniguchi Y, Choi PJ, Li GW, Chen H, Babu M, Hearn J, et al. Quantifying E. coli proteome and transcriptome with single-molecule sensitivity in single cells. Science. 2010 7;329(5991):533–538. Available from: https://science.sciencemag.org/content/329/5991/533.

24. Wang M, Weiss M, Simonovic M, Haertinger G, Schrimpf SP, Hengartner MO, et al. PaxDb, a database of protein abundance averages across all three domains of life. Molecular & Cellular Proteomics. 2012 8;11(8):492–500. Available from: https://www.sciencedirect.com/science/article/pii/S1535947620329947.

25. Drummond DA, Wilke CO. Mistranslation-induced protein misfolding as a dominant constraint on coding-sequence evolution. Cell. 2008 7;134(2):341–352. Available from: https://doi.org/10.1016/j.cell.2008.05.042.

26. St-Pierre F, Cui L, Priest DG, Endy D, Dodd IB, Shearwin KE. One-step cloning and chromosomal integration of DNA. ACS Synthetic Biology. 2013 Sep;2(9):537–541. Available from: https://pubs.acs.org/doi/10.1021/sb400021j.

27. Kaiser M, Jug F, Julou T, Deshpande S, Pfohl T, Silander OK, et al. Monitoring single-cell gene regulation under dynamically controllable conditions with integrated microfluidics and software. Nature Communications. 2018 1;9(1):212. Available from: https://doi.org/10.1038/s41467-017-02505-0.
