# Supplementary material for: Genome-wide gene expression noise in Escherichia coli is condition-dependent and determined by propagation of noise through the regulatory network
Source: PLoS Biol. 2021 Dec 17;19(12):e3001491. doi: 10.1371/journal.pbio.3001491 (PMC8719677; doi:10.1371/journal.pbio.3001491)
Supplement: Attachment — Submitted filename: response_reviews_25sep2021.pdf [file pbio.3001491.s003.pdf]

Response to the reviews of PBIOLOGY-D-21-01107R1: Genome-wide gene expression noise in Escherichia coli is condition-dependent and determined by propagation of noise through the regulatory network

Basel, September 22, 2021

Dear editors,

Below are our point-by-point responses to all the issues raised by the reviewers, which explain all the new materials and analyses in the revision that address each of these points. We were generally very pleased with the constructive comments of the reviewers which enabled us to significantly improve the manuscript. Before getting to the detailed responses, we would like to summarize the main additional experiments, analyses and results that we have added in the revision. These include:

1. From the comments of reviewer 1 we realized that, in an effort to make the presentation of this work self-contained, we failed to properly lay out its relationship with a previous publication from our lab [1], and this has caused confusion. In response we have significantly rewritten the manuscript to properly explain how the current work was motivated by the previous work of [1].

2. In response to comments of both reviewers 1 and 2, we have now included additional analysis about the role of fluctuations in cell size (using data from our microscopy measurements of single-cell sizes in each condition). We find that the coefficient of variation of cell size is similar in each condition and does not correlate with either growth-rate or the noise floor. Consequently, we conclude that the dependence of the noise floor on growth-rate is not related to changes in the cell size distribution (Suppl. Fig. S9).

3. In response to comments of reviewer 1, we have made significant changes to Figure 2. We replaced one Figure panel with a new panel and added a Supplementary Figure (Suppl. Fig. S11) that shows how average expression, expression plasticity, average noise, and noise plasticity vary across the entire set of promoters.

4. In response to comments by reviewer 2, we have performed new experiments in which we directly compare our plasmid-based reporters with chromosomal integrations of the same reporter constructs. Our reporters use a low copy-number plasmid and we find that, for these plasmids, copy-number fluctuations are *not* a significant source of additional expression noise. In particular, we find that the measured noise levels of promoters near the noise floor are very similar for the plasmid-based reporters and corresponding chromosomal reporters (Suppl. Fig. S10).

5. In order to more directly show that constitutive promoters are consistently low noise across conditions we performed new experiments in which we measured noise levels across conditions for a selection of constitutive synthetic promoters (obtained previously in [1]). These results are shown in Suppl. Fig. S15.

6. Similarly, to support more directly that high noise promoters are all regulated, we added a panel to Fig. 4 showing the fraction of high noise promoters that have at least one known

regulatory input, as a function of the cut-off on noise level (Fig. 4B and Suppl. Fig. S16). Even though current knowledge of *E. coli*'s regulatory network is very incomplete, with known targets for only about 1/3 of TFs (see also Suppl. Fig S14), we find approximately $70-90\%$ of high noise promoters in each condition have at least one known regulatory input.

7. To address questions about the statistical significance of the results in Fig. 4A, we have added new analysis (Suppl. Fig. S13) directly reporting t-statistics for these results, confirming that these results are highly significant.

Apart from these we have substantially rewritten the text in many places to clarify issues raised in the comments of the reviewers. We feel that these changes have substantially strenghtened our paper and thank the reviewers for their useful comments. We believe that our revisions fully address all points raised by the reviewers and hope that you will now find our manuscript acceptable for publication. If any points remain unclear, or if there is any additional information or clarification that we could provide, please do not hesitate to contact us.

Sincerely,

Erik van Nimwegen

Detailed responses to the comments of reviewer #1

Comment 1.1

This is a well-reasoned and well-presented manuscript studying the role of environmental conditions in expression variability in E.coli. The authors hypothesize that a key factor in expression noise is that genes regulated by transcription factors will propagate the noise of the factors and will therefore be more noisy the factors or the number of factors. The ultimate conclusion, that environment affects noise through transcription factors and that it affects different genes differently, is not surprising.

This manuscript builds heavily upon this group's previous work. The manuscript is well written, and the clarity of data analysis and presentation of supporting evidence is exemplary. Overall, the manuscript is impressive in my opinion has the potential to support many interesting developments, both theoretical and experimental. I do have, however, several important concerns that should affect the editorial decision. Once these major issues are addressed, I'll be happy to review the manuscript again.

Response 1.1

We thank the reviewer for these comments. Below we respond to the specific comments of the reviewer and explain how we have addressed her/his concerns in our revision.

Comment 1.2

1. Some of the results presented are quite similar to the senior author's older work "Expression noise facilitates the evolution of gene regulation" (Wolf et al., eLife 2015). Indeed, the following quote is from that eLife 2015 publication digest entry: "Wolf et al. also found that noisy promoters tend to be highly regulated by transcription factors: the proteins that control gene expression by binding to promoter regions." This sounds much like the take-home message of this manuscript, which raises questions regarding the contribution in this manuscript.

Response 1.2

Rereading our presentation we understand why some confusion may have arisen. We wrote our manuscript focusing on providing a self-contained presentation of the results and the logic of the approach, but we now realize that the connection of this work with our previous work [1] had become unclear. It is certainly true that the work we present here is to a large-extent motivated by the results that we obtained in [1]. However, the work presented here is an entirely new project and the approach, main results and insights that we obtained are all new.

In [1] we used in-lab evolution to evolve synthetic promoters from random sequences and then compared the noise properties of these synthetic promoters with those of native promoters for bacteria growing in minimal media with glucose. Observing that almost all synthetic promoters were low in expression noise, and observing that more noisy native promoters tended to have more known regulatory inputs, we derived the hypothesis that noise propagation was an important determinant of gene expression noise. However, the emphasis of the paper [1] was on the development of a theory to explain why noise propagation could be evolutionarily beneficial under quite general conditions.

As a result of these previous findings, we were motivated to systematically investigate experimentally to what extent noise propagation drives expression noise on a genome-wide scale. A key characteristic that distinguishes noise propagation from other sources of expression noise is that this noise is *condition-dependent.* We thus decided that a systematic investigation of the condition-dependence of genome-wide noise patterns in *E. coli* is the most direct way to study the role of noise propagation genome-wide. Importantly, there has so far not been any systematic investigation of how gene expression noise varies across conditions, so investigating genome-wide expression noise across different growth conditions is of interest in its own right.

The main results of our study are:

1. We provide a systematic quantification of noise levels genome-wide across conditions (which provides a valuable resource for researchers in the field).

2. We observe that noise levels can be separated into a promoter-independent part that sets a condition-dependent 'noise floor' below which no promoters go, and a highly promoter-dependent excess noise on top of this noise floor.

3. The noise floor decreases (approximately linearly) with the growth-rate of the cells and the overall variation in noise levels decreases with growth-rate as well.

4. Constitutive promoters exhibit low noise in all conditions, and show low plasticity in their noise levels.

5. In each condition, the higher the noise of promoters, the more known regulatory input they tend to have.

6. The more regulatory inputs promoters have, the higher is the plasticity in their noise levels across conditions.

7. Individual promoters show highly distinct changes in their noise levels across conditions.

8. In spite of our very incomplete knowledge of *E. coli*'s regulatory network, a very crude model of noise propagation captures a significant fraction of the condition-dependent variation in noise levels and identifies TFs that propagate noise in specific conditions.

9. A systematic study of the correlation structure of genome-wide variation in evolutionary and gene expression properties of genes shows that genes are organized along two independent axis: the first organizing genes by mean expression level, codon bias and evolutionary rates, and a second independent axis organizing genes by regulatory inputs, expression plasticity, average noise, and noise plasticity. Moreover, these latter quantities are all positively correlated genome-wide.

With the exception of the observation that more noisy genes tend to have more regulatory inputs (which we made in [1] and we now extend to multiple conditions) all these results are new. In our revision we have rewritten the presentation so as to make the relationship of these results to the work in [1] clearer.

Comment 1.3

Unfortunately, the authors have made my life very difficult as a reviewer, by blending old and new methods in their manuscript in a way that makes it difficult to assess the novelty in this work. For example: most of Fig. 1 in this manuscript is very similar to Fig. 1 of Wolf. Fig. 2B in this manuscript is very similar to Fig. 1E in Wolf. Fig. 4A in this manuscript is very similar to Fig. 2A in Wolf. Fig. 4B in this manuscript is very similar to Fig. 2C in Wolf. There are more examples. The fact that as deep as Fig. 4 in this manuscript I still see analyses that are very similar to this group's work from 6 years ago, is worrying. It suggests to me that the authors need to rewrite the manuscript in such a way that makes it utterly clear what the novelty in this work. This would be a major edit since it is expected that non-novel methods/analyses belong either in the introduction or in the supplementary material, and that the results section contains only the novel results for this work. Only after the authors have clearly separated the old from the new, will I be able to properly judge the significance of the new manuscript. As it stands currently, my impression is that the major breakthrough was in the 2015 paper and that this followup work simply implements those methods in more experimental conditions.

Response 1.3

While, as indicated in our last response, we agree with the overall comment of the reviewer that we needed to better explain the relationship of the results in this paper with those of [1], these claims about figure simularity are unjustified. In particular

- Figure 1 of this manuscript is not 'very similar' to Figure 1 of [1]. Figure 1 of [1] is almost entirely focused on explaining the in-lab evolution experiments (panels A through C) and on showing that noise levels in synthetic promoters are lower than in native promoters (panel E). In contrast, the focus of Figure 1 of our current manuscript is on explaining how we measured noise levels genome-wide across conditions (panels A and B), and showing how noise distributions and the noise floor vary across conditions and with growth-rate (panels C and E).

  It is true that panel 1D of [1] and panel 1B of our current paper are very similar, i.e. they both show a scatter of mean and variance in log-expression across promoters (although the results shown are of course different!). But the occurrence of two similar looking figure panels is just because in both papers we have measured noise levels genome-wide with flow cytometry, and we want to illustrate the overall form that the results of such measurements take. We think it is hardly avoidable to include such a panel if our manuscript is to be self-contained.

- It is not true that Figure 1E in [1] and Figure 2B of the current manuscript are very similar. In fact, there is no similarity whatsoever between these figures.

- Figure 2A of [1] and Figure 4A in this work are not very similar either, i.e. they show fundamentally different results. We presume the reviewer was probably thinking of Figure 2B of [1], which we agree has some similarity with our Figure 4A, i.e. they both show the association between regulatory inputs and noise levels across promoters. However, Figure 4A is not repeating the results of Fig. 2A of [1]. The purpose of Figure 4A (and the associated figures in the supplement) is to show that the observation that we made in Figure 2B of [1] *generalizes* to noise levels across multiple conditions. We agree we should have made this clearer and in the revision we have now added remarks to make sure this is clear.

- Figure 2C of [1] and Figure 4B of the current work (now Fig. 4C) may look similar visually, i.e. they compare cumulative distributions across promoter classes. But they show fundamentally different results. Figure 2C in [1] compares noise levels in M9 glucose of different classes of promoters while Fig. 4B (now 4C) in the current manuscript looks at the distribution of *noise plasticity*, i.e. how variable noise is across different conditions. This is a completely different result which requires comparison of noise levels across conditions.

Finally, in response to the comment about non-novel methods, we would like to stress that neither [1] nor our current paper are about methods. It is true that a key experimental methodology, i.e. measurement of transcriptional noise using fluorescent reporters in combination with flow cytometry, is used in both [1] and in our current manuscript, but this is not the topic of the paper. Notably, such approaches were already used in earlier papers such as [2], which is cited in our manuscript. We have of course worked on improving these methods, especially with respect to the computational analysis of such flow cytometry data, but this is also not the topic of this paper. As we indicate in the manuscript, we have already reported on these improved computational methods in [3].

Comment 1.4

2. I think the manuscript can be made more robust by softening some of the interpretations the authors make about the data. The data as they are speak eloquently and I fear these particular interpretations may detract and even may misguide and so should be removed.

** Fig 1C - there not enough data to justify a linear fit. These data could be explained by a number of plausible models, for example a Hill function. Similarly, the interpretation in the text is not necessary. Unless the authors suggest a plausible linear relation, and then extract the slope of the linear fit and discuss its implications (adjusting for the correct units and considering fit confidence levels) then I suggest the authors do away with this fit altogether.*

Response 1.4

We agree with the reviewer that the description in the text was too forceful and might have suggested that we somehow have a *model* for why the noise floor should decrease linearly with growth-rate. In the revision we have changed the text so as to make clear that we are just reporting what the data show and do not have a theoretical argument for why the relationship should be linear.

But we do not see what's wrong with perfoming a simple linear fit. This fit is just to show that the data almost perfectly fall on a straight line, i.e. with a squared Pearson correlation of 0.96, indicating that 96% of the variance is captured by this simple linear fit. Also, the data in

Fig. 1C cover essentially the entire range of possible growth-rates for *E. coli* from no growth at all to fast growth with roughly 25 minutes doubling time. That is, it is not the case that this linear dependence only applies to some limited range of growth rates.

Finally, we do not understand why the reviewer suggests fitting a Hill function. A Hill function is an S-shaped function that is considerably more complex than a straight line and given that the data is fit so well with a straight line, it makes little sense to us to fit a more complex relationship unless there were a plausible theoretical reason for why the noise floor as a function of growth-rate should follow a Hill function. But we cannot think of any such theoretical reason.

Comment 1.5

** Since Growth rate ∼ size and noise floor ∼ 1/sqrt(size), doesn't that imply that the faster the growth the lower the noise? (e.g. Fig. 1C). This seems like an obvious statement, and so should be made clear. Again it questions the validity of the linear fit in Fig. 1C.*

Response 1.5

It is true that average cell volume increases with growth-rate (indeed this is shown in Suppl. Fig. S3) but the reviewer seems to be mixing up the noise floor with a Poisson component of the noise. As we explained in the manuscript and detailed in section S1.1.5 of the Supplementary Information, the noise floor corresponds to a minimal amount of variance in *log expression*. This is equivalent to a minimal coefficient-of-variation (the ratio of standard-deviation and mean) and corresponds to an expression variance that grows proportional to the *square* of mean expression. Thus, in contrast to the Poisson noise component, which contributes a term proportional to $1/G$ to log-expression variance for a gene with mean expression G, the noise floor does not depend on absolute expression level. Indeed Fig. 1B shows that at high expression levels, where the Poisson component has become negligible, the lower bound on noise no longer decreases with mean G but converges to the noise floor. The noise floor is thus not dependent on the absolute value of the expression and there is consequently no reason to expect the noise floor to decrease with cell size.

However, the reviewer's remarks made us realize that we should have included a discussion regarding the possible effect of cell size fluctuations on the noise floor. In particular, while rescaling of the average cell size will not affect the noise floor, the amount of cell-to-cell variability in cell size could plausibly affect the noise floor. For example, if the variation in cell size were to decrease with growth-rate, that could explain the observed decrease in noise floor with growth-rate (at least partly). Therefore, for the revision we have included a new analysis where we calculated the coefficient-of-variation (CV) of cell size for each growth-condition using our microscopy data, and investigated whether this CV depends systematically on growth-rate. However, as shown in Suppl. Fig. S9 of the revision, the CV of cell size varies relatively little across conditions, and neither the noise floor nor the growth-rate shows any systematic correlation with the CV of cell size. These results thus argue that fluctuations in cell size are not the main determinant of the change in noise floor with growth-rate and we have added corresponding remarks about this in the revision.

Comment 1.6

** Fig 2E-F - I'm not sure the evidence supports the statements - "Noise/Expression plasticity". How different are they from A-B? One outlier is hardly convincing. Do the authors really want*

to go into the statistical analysis of whether they are or aren't different from the A-B case? Please remove/adjust or otherwise support the statements with a rigorous statistical analysis.

Response 1.6

The main purpose of Fig. 2 is to show that individual promoters exhibit highly individual behavior in noise and mean across conditions. We now realize that, because of the way we presented the data in Fig. 2, a reader might have come away with the impression that we were claiming there are well-delineated categories of behavior. This was certainly not what we intended to suggest. On the contrary, there is in fact an almost continuous range of different behaviors across promoters.

For the revision, we changed Figure 2, and added a supplementary figure (Suppl. Fig. S11) to make this clear. Figure 2A now shows a scatterplot of plasticity in mean expression against plasticity in expression noise across all promoters, showing that there is a wide and approximately continuous range in behaviors across promoters, and no systematic correlation between these quantities. In Suppl. Fig. S11 we show analogous scatters comparing average expression, expression plasticity, average noise, and noise plasticity across all promoters. As we stress in the text of the revision, these scatters show that there is a wide range of behaviors across promoters, without strong systematic dependence between these variables, and we explain that the figure panels 2B through 2G simply show some examples of the different behaviors that different promoters exhibit.

As to the comment about statistical analysis. We believe that we are actually quite careful with our statistical analyses and would like to point out that every datapoint in figure panels 2B through 2G has error bars for both the value along the horizonal and the vertical axes. Moreover, these error-bars are obtained directly from variability across 6 biological replicate experiments. Therefore, any two datapoints that are clearly outside of each other's error-bars in these panels are statistically significantly different. It is thus certainly the case that the behavior of every promoter shown in panels 2B through 2G is statistically significantly different from the behavior of every other promoter. That is, although we certainly cannot say that there are clearly delineated categories of behavior, we *can* certainly say that all these promoters behave differently.

Detailed responses to the comments of reviewer #2

Comment 2.1

In the manuscript Urchueguía et al study gene expression noise in E. coli using a plasmid-based reporter system and flow cytometry. Using their system, the authors recapitulated what is known about noisy gene expression (variation among genes, increase of noise with lower expression levels, and a minimal noise floor) and expanded it to multiple growth conditions (faster growth reduces the overall noise level). While the choice of methods is tricky, both plasmids copy number is inherently noisy, and so is flow cytometry, the analysis appears well done.

Response 2.1

We thank the reviewer for this comment. The issue of plasmid copy-number comes up below in comment 2.7 and we refer to our response 2.7 regarding this issue. We comment on flow cytometry analysis in response 2.8.

Comment 2.2

Fig. 2 is a nice illustration of the behavior of different promoters and Fig. 3 states the authors hypothesis that noise propagation increases noise clearly. The analysis of the effect of regulation on noise (Fig 4) yielded interesting results. Noisy genes generally had more regulatory input, and highly regulated promoters showed higher noise plasticity.

The authors interpret their findings in Fig. 4 as 'Noise propagation explains condition-dependent noise levels', which at this stage I cannot see that the authors have shown. In Fig. 4C the authors show the fraction of explained variance of their simple noise propagation model, which captures around 10 to 30% of the observed noise. With 90-70% of the observed noise unaccounted for, it is hard to argue that noise propagation has explained variations in noise levels. I believe that is OK for the authors to only explain noise levels partially, and I also believe that Fig. 4A and B are interesting new data.

Response 2.2

It is not correct to assume that, because our simple noise propagation model is only able to explain $10-30\%$ of the variance in noise levels, $70-90\%$ of the variance has to be *caused by something else*. For example, there is currently also no computational model that can successfully model genome-wide variation in *mean* expression levels across conditions in terms of the actions of TFs. But that doesn't mean that TFs are not responsible for regulating gene expression. It just means that current models are very inaccurate (because of a lack of information). The situation is similar for our model of noise propagation.

As we explained in the paragraph below equation (1) in the manuscript, our current knowledge of *E. coli*'s transcriptional regulatory network is extremely incomplete. In particular, for almost two thirds of the TFs essentially nothing is known about which promoters they target and for almost 60% of all promoters no regulatory inputs are known (Suppl. Fig. S14). Moreover, even for the regulatory interactions that we happen to know about, we know virtually nothing quantitatively about how TF levels in single cells affect the transcription rates at individual promoters and how this depends on the binding of other TFs to the promoter. All we know is that, if a known regulatory interaction exists, then fluctuations in the TF may propagate to fluctuations in transcription rate of the promoter but we know nothing about the size or context-dependence of these potential effects. Lacking this information, our model makes the extremely crude assumption that the fluctuations of a given TF have the *same* affect on the noise of all its target promoters, irrespective of what other TFs may target each promoter.

Given these severe limitations, we would not expect this highly simplified model to explain much of the total variance in noise levels, even if *all* expression noise was due to noise propagation. In fact, given how incomplete and rudimentary our knowledge is, we find it remarkable that as much as 30% of the variance in noise levels can be captured by this model.

In the revision we have made sure to explain these points more clearly.

Comment 2.3

Fig. 4D-E are accompanied by a large section in the text (p. 10) explaining which transcription factor is important and noisy for certain reasons in each condition. While some of the claims could be true (flagellar master regulator being turned on at end of growth $\rightarrow$ noisy in early stationary phase), most others are not trivial (ArcA upregulated at high salt, because cell turns fermentative. But why the noise?). This section is very speculative, and could leave the reader more confused than enlightened. I recommend removing it from the main text.

Response 2.3

We agree with the reviewer that this discussion is speculative and in the revision we have moved much of this discussion to the Supplementary Information. However, we feel it is important to highlight that our simple model makes concrete predictions about which TFs are most responsible for propagating noise in individual conditions and have left in a short discussion of particularly plausible cases (e.g. LexA in cipro and FlhDC in early stationary phase).

Comment 2.4

The authors should make clearer what the analysis of regulators (transcription factors and sigma factors) found means for the noise levels. In Fig. 4E the average noise propagation strength for a 'hit' is 0.01, but it was not clear to me if this is little or a lot. After going through the manuscript again I now understood that a gene regulated by this TF/sigma increases its noise level by approx. 0.01 (Eq. 1). Because the typical scale is 0-0.15 in noise level for a gene (Fig. 1D), regulation increases noise by around 10%. A proper estimation of what the numbers mean could benefit the reader.

Response 2.4

As discussed in the manuscript, and in response 2.2 above, because our knowledge of *E. coli*'s regulatory network is so incomplete and rudimentary, we do not expect this very crude model to be able to get anywhere near correctly predicting the noise levels of the promoters. This is not the aim of the model. The aim is to confirm that noise propagation can explain a significant amount of the variation in noise levels across conditions, and to identify which TFs are most significantly propagating noise in each condition. As such, the numerical values of the noise propagation strengths for different TFs are not relevant for our aims, and because their interpretation is actually rather subtle, we avoided higlighting them on purpose. From the reviewer's comments we now understand this was a mistake and in the revision we have included some remarks regarding the interpretation of the noise propagation strengths.

The noise propagation strength of a TF in a given condition corresponds to the *average* amount by which the noise of targets of the TF is *predicted* to increase due to noise propagation. Although we understand that it is tempting to think of this quantity as the typical amount of noise that the TF adds to each of its targets, this is not correct and actually quite misleading.

In a given condition C, different target promoters of a given TF X will respond to different extents to fluctuations in binding of X. For example, target promoters of X that are tightly repressed by another TF will just not be expressed and not respond to binding/unbinding of X at all. At the other end of the spectrum, one may have promoters that are already transcribing at a maximal rate because of another activating TF, and these will also not respond to fluctuations in X. So no noise will be propagated to these targets of X. In addition, in condition C the concentration of TF X will fluctuate in some typical range and the extent to which a given target promoter will experience fluctuations of the binding of X will depend on the number and affinities of its target sites. That is, some target promoters may have such strong sites that they are essentially bound all the time, independent of the fluctuations in the concentration of X, and some others may have such weak sites that they are essentially never bound. Such promoters will also not experience significant noise propagation from X. The only targets of X that will experience significant noise propagation from X are those for which the binding of X significantly affects the transcription rate in condition C, *and* the strengths of their binding sites is such that the binding of X fluctuates significantly.

However, lacking any information about such details, our model cannot predict which targets will respond more or less strongly to fluctuations in X, and so the model just predicts that all targets will respond by the exact same amount. Since this is almost certainly incorrect, and only a fraction of target promoters will actually be responsive, the fitted noise propagation strength is a conservative trade-off that balances correctly predicting noise propagation to those promoters that are responsive in condition C, against incorrectly predicting a response in the other target promoters that are not responsive in condition C. Thus, if only 10% of the targets are responsive in condition C, and their noise levels are *fully* explained by noise propagation from X, then the model would still fit a noise propagation strength corresponding to 10% of their noise levels, because the other 90% of the promoters are not responsive. Of course, the same noise propagation strength might correspond to explaining 50% of the noise of a subset of 20% of sensitive promoters, and so on. But it is almost certainly not the case that the noise propagation strength corresponds to explaining 10% of the noise levels in all its targets.

Since these points were unclear, we have added a discussion about these points in the section on the expected effects of noise propagation, and also refer to them in the section describing the results of the noise propagation model.

Comment 2.5

Finally, Fig. 5 seems ok, but very confusing and needs more explanation. What are dS, dN? I assume codon bias of synonymous and non-synonymous sites, but it is not clear to me what the metric is. What does it mean that genes cluster along the two principal components? Is this trivial, because several noise metrics were included in a data set where the other metrics are highly correlated (protein, RNA expression, dS, dN), or is it non-trivial? Is the conclusion that noise is independent of expression level? What does it mean that regulatory input is only a small component of PCA2?

Response 2.5

We accept that we failed to clearly explain Figure 5 and have updated its description in the revision. The aim of this analysis is to elucidate the overall genome-wide correlation structure of this set of 10 gene features. dS and dN measure the rate of substitution of the gene at synonymous and non-synonymous sites, i.e. these statistics measure evolutionary rates, which we now mention explicitly in the text.

Importantly, contrary to what the reviewer suggests, almost none of these features show high correlations. The highest correlations are between dN and dS, and between average RNA and protein levels, but even those are not close to 1 in Pearson correlation. Moreover, although it is well-known that dN and dS correlate negatively with protein and RNA levels [4–6], these correlations are fairly weak, i.e. with a squared Pearson correlation below 0.1 (which, for comparison, is less than the fraction of variance that our crude noise propagation model explains).

The first main finding of this analysis of the correlation structure is that more than 50% of the variance in this 10-dimensional space lies in the plane spanned by the first two principal components. This says that the majority of the variance in these features across genes can be captured by just two axes.

We note that, by construction, these two principal axes are orthogonal, i.e. the variation along one axis is statistically independent of variation along the second axis (which we now point out explicitly). The second and most interesting observation is that the first PCA axis aligns almost entirely with features associated with mean expression and evolutionary rate, whereas the second axis aligns almost entirely with features associated with regulation, expression plasticity, and noise. That is, we indeed find that features associated with evolutionary rates, codon

bias and mean expression vary independently from features associated with gene regulation and expression noise (as we now stress much more explicitly).

Finally, the exact values of the weights in Fig 4C and 4D are difficult to interpret because they result from a combination of how significantly each feature varies in the first place, and how well this variation aligns with the PCA axes. We suspect that the number of regulatory inputs has smaller overall weight because this feature is the most noisy and incomplete, but this cannot be confidently concluded with the information that we have. In any case, the precise values of these weights are not relevant for the interpretation of these results.

Comment 2.6

Overall, the manuscript is fine and can be published after revisions. In addition to the above comments, I want to express a concern that that the claims of the authors, especially in title and abstract, are not fully supported by the data.

Expression noise, as far as I can see, is only 'partially determined' by noise propagation (10 to 30%, if I understand the paper right), so the title should be adapted to reflect that. The abstract should include that instead of a 'significant fraction' of the variation in noise levels, about 10-30% can be explained by the authors. Being transparent on the findings is important, because it will leave other authors (or the same) the opportunity to continue to work on what determines the remaining 70-90% of the noise.

Response 2.6

We are happy to read that the reviewer feels the manuscript should be published upon revision.

Our title does not claim that *all* of gene expression noise results from noise propagation. Indeed, our results also show that there is a condition-dependent lower bound on noise, and that both the genome-wide mean and variance in noise levels decreases with growth-rate. These genome-wide trends do not result from noise propagation. What we claim is that we observe that relative noise levels are highly condition dependent, and that noise propagation is a major determinant of this condition-dependent noise.

As we explained in the responses above, the fact that our very crude model of noise propagation captures only 10-30% of the variance in relative noise levels does not imply that noise propagation is only responsible for 10-30% of the variation in noise levels. In fact, given our highly incomplete knowledge of *E. coli*'s regulatory network, the true fraction is likely much higher, and we feel it would be misleading to suggest that we can meaningfully estimate what fraction of noise levels is really due to noise propagation.

Moreover, we want to stress that our claim that noise propagation is a major determinant of condition-dependent noise levels is not only supported by the fits of this very simple noise propagation model. Figure 2 shows that different promoters show very diverse behaviors in mean and noise across conditions. This is expected under a noise propagation scenario, but hard to explain if transcriptional noise results from a generic property of the promoter. In fact, we have difficulty thinking of another plausible explanation for the highly individualistic condition-dependent behaviors of different promoters. Figure 4 shows that genes that have higher noise on average systematically have higher numbers of regulatory inputs, and that the more regulated a gene is, the higher the plasticity of its noise tends to be. Figure 5 shows that, when analyzing the correlation structure of gene features genome-wide, the second PCA axis corresponds to average noise, noise plasticity, expression plasticity, and regulatory inputs, and that all these features are positively correlated on a genome-wide scale. In addition, as we detail below, in the revision we now also include new results to show that constitutive promoters have consistently low noise, low noise plasticity, and low expression plasticity, and that $70-90\%$ of high noise

promoters have at least one known regulatory input. We submit that all these results together make a convincing case that noise propagation is a major determinant of condition-dependent noise levels.

Comment 2.7

Furthermore, I want to authors to state in the abstract that their system is plasmid-based, because plasmid themselves are highly noisy, and the reader needs to be aware of this limitation of the study. In the main text, the authors should include a discussion of plasmid copy number variations and compare the noise from plasmid copy numbers to their observed noise (e.g. see https://www.nature.com/articles/s41467-021-21734-y).

Response 2.7

We agree with the reviewer that we should have discussed potential effects of using a plasmid-based reporter. However, we like to stress that the *only* difference between the reporter constructs for different promoters is the inserted promoter sequence. Therefore, plasmid copy-number variations will affect all reporter constructs in the same way and differences in mean expression or expression noise across promoters can only be caused by differences in the promoter sequence, i.e. they almost certainly reflect differences in transcription. Since the large majority of our results depends only on these relative noise levels, these results are unaffected by plasmid copy-number noise. We have now included comments to stress this point.

However, we agree that the observed noise floor could be affected by plasmid copy-number variation. That is, it is conceivable that the observed decrease in the noise floor with growth-rate could reflect a decrease in plasmid copy-number fluctuations with growth-rate. There is a common belief in the field that noise in plasmid copy-numbers is a major contributor to gene expression noise. However, it important to realise that plasmid copy-numbers highly depend on the origin of replication, and that the different plasmid origins of replication (e.g. colE1, pUC, pBR322, pSC101) likely exhibit very different noise levels in copy-number.

We thus decided to perform new experiments for the revision to directly address the possible role of copy-number noise for the plasmid that we employ (the low copy-number plasmid reporter system based on the pSC101 origin of replication). We selected a set of 5 native promoters that were observed to have noise near the noise floor across all conditions. In addition, in our previous study [1] we obtained synthetic promoters by screening a library of random $100-150$ bp sequence fragments and most of these promoters also exhibit consistently low noise. We additionally selected 4 synthetic promoters with low noise. Finally, we also selected one synthetic promoter with relatively high noise. We created chromosomal reporters for all 10 of these promoters, and systematically compared mean and variance in log-expression of these chromosomal constructs with the corresponding plasmid-based reporters.

Unfortunately, the chromosomal reporters have considerably lower expression than the corresponding plasmid-based reporters, and many of these low noise promoters had too low absolute expression to allow accurate quantification of their noise using flow cytometry. We thus decided to measure both the plasmid-based and corresponding chromosomal constructs using microscopy, growing cells in a microfluidic setup as we have described previously [7, 8]. The results are shown in Suppl. Fig. S10 in the revision. They show that mean expression levels of plasmid and reporter constructs are almost perfectly correlated, and plasmid-based reporters have about 6.5-fold higher mean expression. However, the noise levels of the plasmid-based and chromosomal constructs are very similar, with differences generally within the error-bars. These results show directly that, either plasmid copy-number noise is similar to the chromosomal copy-

number noise, or that the copy-number noise of both plasmid and chromosome is simply small compared to other factors that determine the noise floor.

As an aside, we note that, consistent with these findings, in the paper that the reviewer cites the reported copy-number variation for plasmids with the pSC101 origin is in fact fairly low.

We thank the reviewer for raising this point about plasmid copy-number fluctuations as we believe the new experimental results we obtained for the revision have significantly strengthened the paper.

Comment 2.8

A discussion of the impact of cell size, would also benefit the reader. In exponential growth, cells post- and pre-division vary by a factor of 2, which might impact the noise recorded by the authors. The impact of the flow cytometer on noise levels would also be important for the readers to judge noise levels.

Response 2.8

We agree with the reviewer that we should have discussed the possible role of cell-size fluctuations and note that reviewer 1 also raised this point. For the revision we have now included an analysis of the coefficient of variation of cell sizes across conditions. We find that CV of cell size only varies moderately across conditions (i.e. with about a 60% change between highest and lowest value) and that this CV neither correlates with growth-rate, nor with the noise floor (Suppl. Fig. S9). Thus, although cell size variation will contribute to the noise floor, this contribution is relatively constant, and the observed change in the noise floor with growth-rate is not driven by a change in the variability of cell size. This too we believe is a valuable addition to our manuscript and we thank the reviewers for raising the issue.

Regarding flow cytometer noise, we note that we studied this issue in detail and have reported on the results of these studies in a separate manuscript that was recently published [3]. We have now mentioned this more clearly in the revision.

Comment 2.9

The statement: "Whereas constitutive promoters exhibit noise near this 'noise floor' in all conditions [...]" confused me. Does it refer to Fig. 4A, which shows that less noisy promoters are regulated on average by fewer inputs? If so, then the authors have only shown that promoters with lower noise are usually constitutive promoters. Replotting the existing data as number of regulators vs. noise, and then specifically discussing constitutive promoters should be sufficient to test if indeed unregulated promotors are near the noise-floor for in all conditions. Similarly, the statement "regulated promoters are systematically more noisy" should be changed to 'noisier promoters are regulated by an average higher amount of inputs', or alternatively, the can replot their data.

Response 2.9

We agree with the reviewer. We now realize that we actually never explicitly showed that constitutive promoters have consistently low noise across conditions. For the revision we have now obtained new data and included new analysis (Suppl Fig S15) to show directly that constitutive promoters not only have low expression plasticity (i.e. little change in mean expression across conditions) but also have low average noise and low noise plasticity (i.e. their noise is low in all conditions).

Note that one challenge in systematically comparing noise properties of constitutive and regulated promoters is that our knowledge of *E. coli*'s regulatory network is so incomplete. For almost 60% of all promoters, no regulatory inputs are known (Suppl. Fig. S14), but since we only know targets for about one third of all TFs, it is highly unlikely that all these promoters are truly constitutive. Therefore, to obtain a set of promoters that are constitutive with high probability we randomly selected a set of synthetic promoters from the collection that we obtained by in-lab evolution in [1]. These promoter sequences essentially correspond to random 100-150bp fragments that were screened for their ability to drive gene expression in M9 minimal media with glucose, and we showed in [1] that most of these promoters have low noise. For the revision, we have now measured the single-cell expression distributions of these synthetic promoters across 4 different conditions using flow cytometry. As shown in Suppl. Fig. S15, these promoters not only have low expression plasticity (confirming that most of them are likely constitutive) but also low average noise and low noise plasticity.

In addition, in the revision we have also made changes to the way the results are phrased to make sure the claims match what the data show.

Comment 2.10

In addition, a correlation plot, ideally as part of Fig. 4, between noise/noise plasticity and number of regulatory inputs would greatly benefit the readers. A major issue with the claims of the authors is that it is not clear whether the claims are statistically significant. Inclusion of statistical significance tests concerning correlations between noise and regulatory inputs, should be included in the revision.

Response 2.10

The proposed scatter plots are not very informative because the number of regulatory inputs is so quantized, i.e. almost 60% of promoters has no known regulatory inputs, and an additional 30% has only one or two known regulatory inputs (Suppl. Fig. S14).

With regard to statistical significance, we realize that we did not make sufficiently clear that the error-bars in Figures 4A and Suppl. Fig. S12 allow one to directly assess the statistical significance of these results. That is, because the error-bars in these plots correspond to the standard-errors of the means, one can directly read off from the curve how many standard-deviations the number of regulatory inputs of promoters with noise over some cut-off is away from the average number of regulatory inputs of all promoters.

Nonetheless, to report statistical significance more directly, we have now included an additional supplementary figure (Suppl. Fig. S13) that explicitly shows t-statistics of the difference in mean number of regulatory inputs between promoters with noise over and under a given cut-off in noise level. This figure shows that these differences are highly significant, i.e. with the difference in numbers of regulatory inputs being 4 or more standard-errors over a large fraction of the range.

We note that we already provided statistical significances for the results in Fig. 4C (which was Fig. 4B in the original submission) and we also provide results of significance tests for the new results in Suppl. Fig. S15.

Comment 2.11

The statement: "Our results show that expression noise levels are themselves regulated and determined by the propagation of noise through the gene regulatory network", is, as far as I can see, not shown in the manuscript. Same claim in the discussion: "[...] gene expression noise is

[...] a regulated quantity". If the authors want to make this statement, then they should include a direct test of this hypothesis, e.g. by measuring the noise level in synthetic constructs with a varying number of regulatory inputs, or any other experimental verification.

There are several statements in the manuscript that claim that condition-dependent gene expression noise is explained by propagation of noise through the regulatory network, e.g.

"First, all promoters exhibit a condition-dependent minimal amount of noise, and unregulated promoters typically exhibit noise near this noise floor. Regulated promoters exhibit additional noise that results from propagation of noise through the regulatory network, causing different promoters to show condition-dependent noise levels depending on their regulatory inputs. These results show that not only the mean expression levels of genes are determined by gene regulation, the noise levels of genes are also to a large extent determined by the structure of the gene regulatory network as well." p. 11

The authors should be careful with this claim. If the authors want to make this statement, then they need to show that 1) unregulated promoters are at the noise-floor. 2) only regulated promoters are above the noise floor. I presume that this is based on Fig. 4, but I cannot see which data supports this claim. It could well be that the existing analysis proves the authors point and that I am missing something. Either way, the logic needs to be clearly spelled out and comprehensible.

Finally, it is not clear to me how the authors claim that 'that condition-dependent gene expression noise is explained by propagation of noise through'. This is the major claim of the paper, and is found throughout the paper, including title and abstract. What is the evidence that noise propagation is responsible for the condition-dependent expression noise?

Response 2.11

We feel that these comments suggest that there is a misunderstanding regarding what the main aims and results of our paper are.

Our paper is not about establishing that noise propagation exists. There is absolutely no question in the field that regulators propagate noise to their targets. As we explain in the introduction, the entire mechanistic basis of gene expression regulation is that the binding and unbinding of TFs to promoters affects the transcription rate at these promoters. Consequently, propagation of noise from regulators to their targets is unavoidable and we provide references to over a dozen articles in the literature discussing this. The question is not whether propagation of noise through the regulatory network exists, there is wide agreement that it does. The question is how much this noise propagation contributes to genome-wide transcriptional noise.

That is, it would be conceivable that noise propagation is only a relatively small effect and that noise levels of promoters are completely dominated by other sources of noise. For example, noise levels of promoters could be dominated by some intrinsic features of the promoter that are unrelated to regulation, e.g. its sensitivity to supercoiling, so that each promoter would have a noise level that is largely condition *independent*. Similarly, some previous studies have suggested that the noise levels of promoters are mainly determined by their absolute expression level in a given condition. All these points were discussed in the introduction of the paper.

We also note that the reviewer's suggestion of demonstrating noise propagation in a synthetic promoter does not address what role noise propagation plays on a genome-wide scale. There is no doubt that it is possible to engineer a promoter to respond to the fluctuations in activity of a regulator. In fact, such experiments have already been done. For example, one of the most well-known early papers on noise in gene expression [9] already shows that noise levels respond to changes in the level of a regulating TF (Figure 3B in that paper). But such results for one or a few promoters in one or two artificially manipulated conditions do not tell us much about

the role that noise propagation plays genome-wide across growth conditions.

The aim of our paper is, first of all, to investigate how condition-dependent genome-wide noise levels are, because this has not been systematically investigated. We find that genome-wide noise levels are in fact highly condition-dependent. Second, we find that this condition-dependence can be reasonably separated into a promoter-independent part and a promoter-dependent part. That is, we find there is a condition-dependent lower bound on noise that all promoters are subject to, and this noise floor decreases with growth-rate (Fig. 1C). In addition, the overall variation in noise levels also systematically decreases with growth-rate (Fig. 1E).

Focusing then on the promoter-dependent parts of the noise, i.e. the condition-dependent excess noise in addition to the noise floor, we find that promoters show highly distinct behaviors (Fig. 2). That is, this excess noise varies across conditions in very different ways for different promoters. We then provide a number of lines of evidence to support that noise propagation is a major source of this condition-dependent excess noise. We already discussed these lines of evidence in our response 2.6, but to list them again explicitly:

- The promoter-specific condition-dependence doesn't follow any simple rescaling with growth-rate or absolute expression. However, as we explain in Figure 3, noise propagation naturally explains the observed behavior. We are not aware of any alternative plausible explanation for the highly distinct ways in which different promoters change noise across conditions.

- In all conditions, the higher the noise promoters have, the higher their number of known regulatory inputs (Figure 4A).

- Promoters with more known regulatory inputs have higher noise plasticity (Figure 4C), i.e. the more promoters are regulated, the more their noise levels vary across conditions. We submit that this genome-wide pattern alone already strongly suggests that noise levels are regulated.

- Even though we have only fragmentary knowledge of *E. coli*'s regulatory network, and virtually no quantitative information about the sensitivity of different promoters to noise in the regulators that regulate them, a very crude model of noise propagation still captures up to 30% of the condition-dependent noise levels genome-wide.

- Finally, our PCA analysis of the correlation structure of gene features shows that, on a genome-wide scale, there are two main independent axes along which gene features vary. The first axis is associated with mean expression level, evolutionary rate, and codon bias, and was already well-known [4–6]. The second axis, which we are the first to observe, is associated with expression plasticity, average promoter noise, noise plasticity, and number of regulatory inputs, and these features are all positively correlated on a genome-wide scale. We submit this strongly argues noise propagation is a major determinant of condition-dependent noise on a genome-wide scale.

- Following the reviewer's suggestion, we have now added results that directly show that, indeed, constitutive promoters have systematically low average noise, low noise plasticity, and low expression plasticity compared to promoters with one or more known regulatory inputs (Suppl. Fig. S15).

- In addition, the reviewer asked if it is true that only regulated promoters exhibit high noise. To support this we have added Figures (Fig. 4B and Suppl. Fig. S16) showing the fraction of promoters with at least one known regulatory input as a function of a cut-off on noise level. These results show that, in every condition, $70 - 90\%$ of all high noise promoters have at least one known regulatory input. Given that our knowledge of *E. coli*'s

regulatory network is extremely incomplete, we feel that this in fact strongly suggests that all high noise promoters are regulated.

We are of course not claiming that noise propagation is responsible for *all* promoter-dependent noise, but we do maintain that the above combination of results compellingly support that noise propagation is a major determinant of the variation in condition-dependent noise levels and we believe that our summary of the picture that emerges from our analyses is warranted.

Detailed responses to the comments of reviewer #3

Comment 3.1

This work is a tour de force in characterizing the mean and the variability of E. coli gene expression across many promoters and many experimental conditions. Bravo! This will be a very useful resource for researchers in the field, especially since all of the data have been uploaded by the authors. I am not an experimentalist, and hence cannot assess the experimental parts of the work, which are its cornerstone. The modeling/analysis is rather straightforward, based on previous work from the same group and from other authors, and was easy to follow. I don't have any specific comments there. Based on my ability to understand the computational analysis, and on the beauty of the dataset, I think the manuscript should be accepted to the journal with minimal changes.

Response 3.1

We thank the reviewer for these very kind words.

Comment 3.2

1. As mentioned above, I've been able to follow most of the analysis. However, one argument made me pause. Specifically, the authors say that "Noise propagation predicts that relative noise levels are condition-dependent", so that "genes that are regulated by more TFs are expected to generally exhibit more expression noise than genes that are regulated by fewer TFs". This contradicts the model and the experiments on eukaryotic systems, such as in https://elifesciences.org/articles/59351, which argues that multiple enhancers decrease the promoter variability. There are certainly many differences between eukaryotes and prokaryotes – and it would be worthwhile, I think, for the authors to explain why different results are expected for them under seemingly very similar conditions.

Reponse 3.2

We realize that the sentence that the reviewer refers to can easily lead to a mistaken picture and in the revision we have both edited the presentation, and added additional explanation.

The key difference between gene regulation in prokaryotes and higher eukaryotes is that in higher eukaryotes each gene can be controlled by many distal cis-regulatory elements, which each can contain up to dozens of transcription factor binding sites (TFBSs). The paper that the reviewer cites proposes that, in animals, noise in TF concentrations can be buffered by effectively averaging over the binding at lots of TFBSs occurring in multiple enhancers, with one enhancer 'shadowing' the activity of another. This conjures a picture in which a single gene is affected by the fluctuating levels of many TFs in parallel, in such a way that these fluctuations tend to average each other out, thereby buffering the effect of the fluctuations of individual TF levels.

The structure of prokaryotic gene regulatory networks is very far removed from such a scenario. First, even for a highly regulated prokaryotic gene, there will typically only be a handful or so of different TFs targeting it (Supp.l Fig. S14 of the revision) and only one or two binding sites per regulating TF, all concentrated in a 100-200bp region around the transcription start site of the operon. Second, in a given condition, only a fraction of all TFs will be propagating noise. For example, many TFs will only be activated when a particular nutrient or environmental signal is present, and when these signals are absent the TFs are simply inactive. Other TFs may be highly active so that there are hardly any fluctuations in the effects on their targets, e.g. a given repressor may be expressed at such levels that all its targets are fully repressed. Third, even when a given TF X is noisy in a given condition, a given target promoter Y can easily still be insensitive to the noise from this regulator. For example, if another TF is strongly repressing that promoter, the promoter Y will just be silent, independent of fluctuations in the TF X.

The reason that genes that are regulated by more regulators are more likely to be noisy is not because all its regulators are propagating noise and that this noise 'adds up' to more overall noise. Rather, a highly regulated gene is more likely to be noisy because there is a higher chance that *at least one* of its regulators is significantly propagating noise to it in a given condition.

We have added comments in the revision to make this point clear.

Comment 3.3

2. Towards the end of the manuscript, I got overwhelmed by the data, and was looking for a theoretical framework to incorporate them into, to help internalize them. Why is the organization of regulation as discovered her? Alas, the Discussion section was very short, and, to a large extent, it simply restated the findings of the Results section, without speculations about the framework to understand the data. It might be that the authors do not have a theoretical framework in mind, which I would understand. However, if they do have some ideas, I would strongly urge the authors to put them on paper, to help us summarize the data within some explanatory framework.

Response 3.3

This is a fair comment. For the revision we have made significant changes to the presentation of the PCA results, in an attempt to make them more easily digestible. We have also edited the Discussion session, with the aim of making the general picture that emerges from our results more transparent.

References

1. Wolf L, Silander OK, van Nimwegen E. Expression noise facilitates the evolution of gene regulation. eLife. 2015 6;4:e05856. Available from: `https://doi.org/10.7554/eLife.05856`.

2. Silander OK, Nikolic N, Zaslaver A, Bren A, Kikoin I, Alon U, et al. A Genome-Wide Analysis of Promoter-Mediated Phenotypic Noise in Escherichia coli. PLoS Genetics. 2012 1;8(1):1–13. Available from: `https://doi.org/10.1371/journal.pgen.1002443`.

3. Galbusera L, Bellement-Theroue G, Urchueguia A, Julou T, van Nimwegen E. Using fluorescence flow cytometry data for single-cell gene expression analysis in bacteria. PLoS One. 2020;15(10):e0240233. Available from: `https://doi.org/10.1371/journal.pone.0240233`.

4. Drummond DA, Bloom JD, Adami C, Wilke CO, Arnold FH. Why highly expressed proteins evolve slowly. Proceedings of the National Academy of Sciences. 2005 10;102(40):14338–14343. Available from: https://doi.org/10.1073/pnas.0504070102.

5. Drummond DA, Raval A, Wilke CO. A single determinant dominates the rate of yeast protein evolution. Molecular Biology and Evolution. 2006 2;23(2):327–337. Available from: https://doi.org/10.1093/molbev/msj038.

6. Drummond DA, Wilke CO. Mistranslation-Induced Protein Misfolding as a Dominant Constraint on Coding-Sequence Evolution. Cell. 2008 7;134(2):341–352. Available from: https://doi.org/10.1016/j.cell.2008.05.042.

7. Kaiser M, Jug F, Julou T, Deshpande S, Pfohl T, Silander OK, et al. Monitoring single-cell gene regulation under dynamically controllable conditions with integrated microfluidics and software. Nature Communications. 2018 1;9(1):212. Available from: https://doi.org/10.1038/s41467-017-02505-0.

8. Julou T, Zweifel L, Blank D, Fiori A, van Nimwegen E. Subpopulations of sensorless bacteria drive fitness in fluctuating environments. PLoS Biol. 2020 12;18(12):e3000952.

9. Elowitz MB, Levine AJ, Siggia ED, Swain PS. Stochastic gene expression in a single cell. Science. 2002 8;297(5584):1183–6. Available from: https://science.sciencemag.org/content/297/5584/1183.long.
