# Supplementary material for: Genome-wide gene expression noise in Escherichia coli is condition-dependent and determined by propagation of noise through the regulatory network
Source: PLoS Biol. 2021 Dec 17;19(12):e3001491. doi: 10.1371/journal.pbio.3001491 (PMC8719677; doi:10.1371/journal.pbio.3001491)
Supplement: Attachment — Submitted filename: response_reviews.pdf [file pbio.3001491.s004.pdf]

Response to the reviews of PBIOLOGY-D-21-01107R2: Genome-wide gene expression noise in Escherichia coli is condition-dependent and determined by propagation of noise through the regulatory network

Basel, November 8, 2021

Dear editors,

We were very happy that the reviewers all felt we had satisfactorily addressed their concerns. Reviewer 1 had one final remaining comment (regarding the linear fit shown in Fig. 1C). Below is our response to this final comment and a description of the (small) changes we made to the manuscript in order to address it.

Sincerely,

Erik van Nimwegen

Responses to the comments of reviewer #1

Comment 1.1

The authors have addressed my concerns well.

I appreciate the edits made and personally I find the manuscript much more readable now, and the contribution in this work clearer. The manuscript remains impressive with exemplary analysis practices. I am happy to recommend publication but I do have one comment - I do not need to see the manuscript again - I leave its final form up to the authors and editor.

The authors insist on fitting a straight line to Fig.1C despite acknowledging that they have no theory for it. They then ask me (in their rebuttal) why would I suggest a more complicated form instead. The answer is, that the straight line would go negative for a fast enough growth rate, and simply saying that it's linear within the relevant range for E.coli is hiding that fact under the rug. Together with very sparse data, lacking a reasoned model, such a fit is damaging to the field. This is a good paper in a respected journal - imagine the number of people who would implicitly assume a linear dependence here with little to no justification. Once such a view becomes "lore" it is very difficult to uproot. I request that they remove the ill-argued and quite possibly misleading linear fit from Fig1C. It is not necessary for the main messages of the paper. It is quite enough to state "decreasing" without stating a precise relation. You may even state "approximately linear" or "appears linear" without actually putting the fit with confidence intervals on the figure.

Response 1.1

The reviewer expresses the fear that, in spite of the caveats that we already included, the linear fit of Fig. 1C might still lead to misunderstandings among readers. While we do not share these fears, we have revised the manuscript so as to make sure that such misunderstandings cannot arise.

First, at the request of the reviewer, we have removed the linear fit with confidence intervals and replaced it with a simple dotted line to serve as a guide for the eye. Second, apart from the

sentence which stresses that there is currently no theoretical model to argue that the noise floor must be a linear function of growth-rate (added in the first revision), we have now further added a sentence stressing that the observed linear dependence is nothing more than a phenomenological relationship that applies only to the growth-conditions we investigated, and might not apply to other growth conditions. That is, the sentences in question now read:

"We observed that the noise floor a_c systematically decreases with growth-rate over the entire range of growth-rates. Although we currently lack a theoretical model for how this noise floor depends on growth-rate, we noted that the dependence is well fit by a simple linearly decreasing function ($R^2 = 0.96$, Fig. 1C). However, we stress that this is only a phenomenological observation valid for the growth conditions considered here and that it is currently unclear whether this relationship generalizes to other conditions, e.g. when growth-rate is modulated by sub-inhibitory levels of antibiotics."
